# Bi-Stable Perception: Self-Coordinating Brain Regions to Make-Up the Mind

**DOI:** 10.3389/fnins.2021.805690

**Published:** 2022-01-27

**Authors:** Christ Devia, Miguel Concha-Miranda, Eugenio Rodríguez

**Affiliations:** ^1^Departamento de Neurociencia, Facultad de Medicina, Universidad de Chile, Santiago, Chile; ^2^Biomedical Neuroscience Institute, Universidad de Chile, Santiago, Chile; ^3^Laboratorio de Neurodinámica Básica y Aplicada, Escuela de Psicología, Pontificia Universidad Católica de Chile, Santiago, Chile

**Keywords:** bi-stable perception, neural synchrony oscillations, neural models, multiscale brain activity, EEG frequency bands, brain networks, fMRI, Necker cube

## Abstract

Bi-stable perception is a strong instance of cognitive self-organization, providing a research model for how ‘the brain makes up its mind.’ The complexity of perceptual bistability prevents a simple attribution of functions to areas, because many cognitive processes, recruiting multiple brain regions, are simultaneously involved. The functional magnetic resonance imaging (fMRI) evidence suggests the activation of a large network of distant brain areas. Concurrently, electroencephalographic and magnetoencephalographic (MEEG) literature shows sub second oscillatory activity and phase synchrony on several frequency bands. Strongly represented are beta and gamma bands, often associated with neural/cognitive integration processes. The spatial extension and short duration of brain activities suggests the need for a fast, large-scale neural coordination mechanism. To address the range of temporo-spatial scales involved, we systematize the current knowledge from mathematical models, cognitive sciences and neuroscience at large, from single-cell- to system-level research, including evidence from human and non-human primates. Surprisingly, despite evidence spanning through different organization levels, models, and experimental approaches, the scarcity of integrative studies is evident. In a final section of the review we dwell on the reasons behind such scarcity and on the need of integration in order to achieve a real understanding of the complexities underlying bi-stable perception processes.

## Introduction

Bi-stable and multi-stable perception, also known as perceptual rivalry (Lumer et al., 1998), refers to a process in which insufficient or ambiguous sensory information is provided to the senses such that the perceptual process cannot reach a definitive solution and continues to iterate through two or more perceptual states. This perceptual process provides an opportunity to directly assess the neural dynamics related to perceptual change without following the unecological procedure of physically flashing different stimuli to the visual system of the subject. Bi-stable perception is interesting because using a relatively simple stimulation paradigm allows for the investigation of a variety of neural and cognitive processes, including spontaneous or self-driven changes in brain state (von der Malsburg, 1999), consciousness (Crick and Koch, 1990), the neural basis of self-triggered changes in perception (Blake and Logothetis, 2002), cognitive control of perceptual states (Van Ee et al., 2005), and high-level perceptual gestalt formation (Zaretskaya and Bartels, 2015), among others.

However, despite the scientific interest in this phenomenon, which has resulted in extensive study and a massive number of publications, the neural bases of bi-stable perception are still incompletely understood. Partially, because behavioral, neural and cognitive aspects must be integrated to a complete understanding of this complex phenomenon. Electroencephalographic (EEG) and magnetoencephalographic (MEG) studies have shown the involvement of different brain regions and modulation in several frequency bands, including but not restricted to alpha, beta, and gamma bands (as it will be discussed in depth in a next section). However, as every study has used different methods, stimuli and recording parameters, the exact role of each brain area and frequency band and the mechanisms of their interactions are still unknown.

Despite this incomplete knowledge, a solid assertion can be done so far that bi-stable processes rely on short-lived neural activity, which is widely distributed across brain regions and involves local and long-range coordination over specific frequency bands.

Here, we review the most prominent oscillatory frequencies and related synchronization patterns, along with fMRI localization results and computational models, and present their results in an integrated manner. This is not an exhaustive summary of the literature of the multistable perception field. The contribution of the present review is the complete revision of MEEG experiments reported so far, with a special emphasis on its integration with both behavioral and computational research. Because of the fast and global reorganization of the perceptual field characteristic of bi-stable perception, we make a specific statement regarding the need for methods able to detect fast transient periods of coordinated neural activity. Bi-stable perception requires fast neural coordination across different brain regions and through distinct neural organization scales, the relevant levels include at least neuron-to-neuron, circuit-to-circuit and region-to-region coordination. We propose that such coordination is achieved through transient dynamical coupling based on oscillations, on oscillation synchronization and, likely, on cross-frequency coupling.

## Perception and Bi-Stable Perception

### Characteristics of Visual Multi-Stable Perception

Perceptual rivalry occurs when mutually exclusive perceptions are possible for one physical stimulus. Particularly, perception is called multi-stable when more than two mutually exclusive perceptions are possible and is called bi-stable perception when only two exclusive perceptions are possible (Blake and Logothetis, 2002; Sterzer et al., 2009). Subjects’ perception can switch from one perceptual state to the other mainly without the subject’s control (Sterzer et al., 2009). Evidence shows that under some circumstances, subjects have some degree of voluntary control over perceptual changes, but they also continue to experience spontaneous switches (Van Ee et al., 2005; Klink et al., 2008). In bi-stable perception, the period between two changes in perception is called perceptual stability; the distribution of periods of stability during bi-stable tasks follows a gamma distribution (Zhou et al., 2004). Several factors can modify this distribution (Brascamp et al., 2015b). Motor acts such as eye movements will increase the probability of a perceptual change (Einhäuser et al., 2004); however, even during tasks with gaze fixation, subjects still experience spontaneous changes in perception.

As stated, visual bi-stable perception dissociates perception from physical stimulation (Wang et al., 2008) while also strongly engaging the subject’s attention for extended periods of time, as long as 10 s (Zhou et al., 2004). During perceptual transitions, subjects experience changes in their level of attention but not in the object of attention (Blake and Logothetis, 2002; Sterzer et al., 2009).

### Limitations

The first and main limitation of perceptual rivalry is that the experimenter must rely solely on the subject’s report to know that a change in perception has occurred. This introduces a jitter in the measures given by the time difference between the subjective changes in perception and the subject motor response that can be around 500 ms but can extend to over 1 s in some subjects. Measures can be taken to counterbalance this situation: (1) training the subjects to familiarize them with the motor component of the task to decrease the jitter between reports; (2) estimating the subject’s reaction time to approximate the amount of jitter introduced; (3) using behavioral and physiological measures to complement or replace subject reports, such as eye position or pupil diameter (Einhäuser et al., 2004, 2008); and (4) minimizing the jitter effect by introducing methodological modifications, such as the discontinuous presentation method. The first two measures translate into longer recording sessions, the third measure requires the use of eye tracking, and the fourth is the easiest to implement because it changes only the display images but at the cost of sacrificing the ecological dynamics of continuously viewing bi-stable stimuli.

**FIGURE 1:**
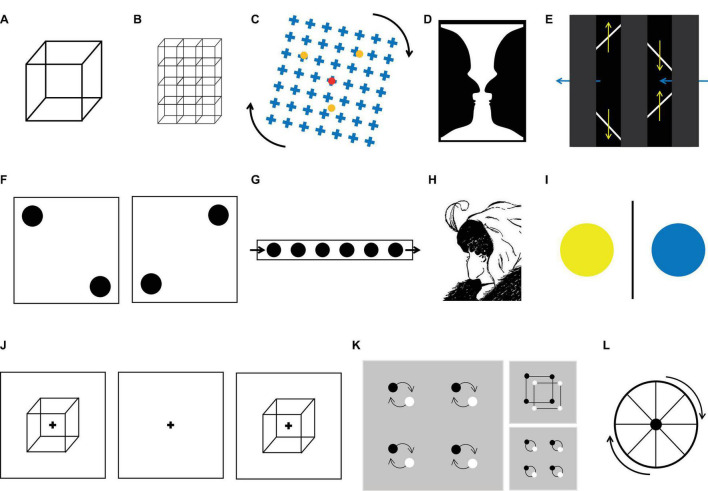
Examples of ambiguous figures. **(A)** Necker cube. **(B)** Array or lattice of Necker cubes. **(C)** Motion induced blindness. **(D)** Rubin vase. **(E)** The diamond-lines illusion. **(F)** Basar dots. **(G)** Moving-dots illusion. **(H)** The lady and old woman illusion. **(I)** Binocular rivalry stimuli. **(J)** Discontinuous presentation method. Here, the bi-stable image was presented (usually for less than a second), followed by a delay, then the presentation of a stable version of the stimulus. The subject’s task was to report if the perception of the second image was the same as that for the first. Trials were classified as perceptual stability or perceptual changes. **(K)** Local vs. global percept illusion. **(L)** Wagon wheel illusion.

The discontinuous presentation method (or onset paradigm) has the advantage of setting an upper limit for the period of time between the actual change in perception and the subject’s report. However, it still has its own limitations. In general, three stages compose each trial of this method (Figure 1J): bi-stability, in which an ambiguous stimulus is presented; delay, in which an empty screen is presented for a period of time; and test, in which either an ambiguous or a non-ambiguous version of the stimulus is shown and the subjects report whether they experienced a change in perception (called reversal or stability, respectively). The duration of the bi-stable and delay stages affects the rate at which perception alternates (Orbach et al., 1963; Orbach and Zucker, 1965; Kornmeier and Bach, 2012), and the duration is adjusted to emulate the dynamics observed during continuous presentation. An upper limit is set because subjects have to report during the test image, and then the delay of the report cannot be longer than the presentation period. The main problem with this method is that it modifies the physical content of the image during the delay period, losing the ecological feature and introducing confounding factors in regard to interpreting the behavior and the brain responses.

A second limitation of bi-stable stimulation is that during perceptual changes, other cognitive functions participate, such as attention, working memory, or expectations, especially during motor responses (Tsuchiya et al., 2015; Brascamp et al., 2018). This is especially important when researching the brain mechanisms that underlie bi-stable dynamics, as the activation of a particular area may reflect the recruitment of those other processes. To control for these other factors, two approaches have been taken: either tracking some non-reported physiological variables such as eye movement or delaying the report to prevent the mixing of perceptual and motor processes (for a more detailed review, see Tsuchiya et al., 2015; Brascamp et al., 2018).

A third limitation in this approach is the difference in the semantic values of the perceptions elicited by the multi-stable stimuli. As perception may differ in some important features, they may evoke different cognitive and brain processes. For instance, when viewing the Rubin vase stimulus (Figure 1D), the subject can perceive either a vase or two faces, which have very different semantic values. In fact, the vase is an inanimate object that correlates with increased activity in the parahippocampal gyrus (Andrews et al., 2002), whereas faces correlate with activity in the face fusiform gyrus (Hasson et al., 2001; Andrews et al., 2002). This is problematic because when averaging trials to calculate the event-related potential (ERP), the semantic value of the stimuli is ignored. To solve this issue, subjects can use two different buttons to report the different perceptions, but authors have found that this can be challenging for subjects as it takes attention away from the task. Another solution to this problem is to use ambiguous images with the same semantic meaning for the two possible perceptions as that in the Necker cube or other moving stimuli (on Figure 1 all except D and H that change their semantic content).

Despite these limitations, perceptual rivalry tasks are currently the best tool to study the mechanisms of self-driven changes in brain state because they induce brain dynamics while maintaining a fixed stimulation.

## Neural Correlates of Perceptual Rivalry

### Computational Models: Implications for Neurophysiology

The neurophysiological bases of perceptual rivalry have not yet been completely established, mainly because this phenomenon has been described mostly in humans though the technology to assess brain activity is limited. Given this current limitation, computational models have been a good tool to infer the physiological mechanisms and the dynamic processes that underpin perceptual multi-stability. Nevertheless, filling the gap between neurophysiological evidence and computational models poses the following important challenges: How is perception represented? What are the relevant features of brain activity for multi-stability? To what extent are models able to predict the existing psychophysical data? Concerning the neural representation of perception, most models assume that during perceptual rivalry, two different neural assemblies compete, and the winner represents the actual perception (Varela, 1995; Blake and Logothetis, 2002); this simple assumption generates a dynamic process that leads to switches between the two neural assemblies (Engel and Singer, 2001), emulating the multi-stability transition process. Regarding the dynamic brain features relevant for multi-stability, models have posited that the most relevant are adaptation, inhibition, and noise because they have a simple computational implementation and a direct physiological interpretation. For example, Kogo et al. (2021), recently developed a hybrid *in vitro* and *in silico* dynamic clamping, where the computational model interacted with neurons in a slice (Kogo et al., 2021). The authors observed that the increase of synaptic noise altered the dynamic of the multistable state, supporting the causal role of noise level on perceptual transitions. Finally, modeling studies have been undertaken to reproduce multi-stability phenomena, as observed in psychophysics studies; however, to date, no single model can explain every psychophysical finding. Through this process, psychophysiological experiments and computational models drive each other, the former guiding the formulation of the models and the latter providing insight into the possible brain mechanism of perceptual rivalry.

The implementation of computational models differentially incorporates features of brain activity, allowing the models to make psychophysical predictions. To date, relevant features comprise membrane potential, firing rate, population activity, noise, adaptation, inhibition, ion conductance, and synaptic depletion, among others (Wilson et al., 2001; Laing and Chow, 2002; Wilson, 2003; Freeman, 2005; Moreno-Bote et al., 2007; Noest et al., 2007); a detailed report of computational models, authors, neural units, physiology, and psychophysics are depicted in Table 1. The first step in modeling is to decide which features will embody perception and which ones will be treated as *latent variables* (see section “Glossary”), that is, variables needed for perceptual multi-stability dynamics but that do not directly reflect a feature of the phenomenon. The second step is to choose the *complexity level* of the model, which refers to the complexity of the interactions between the different features of the model that can be implemented at a particular *spatial scale*. For instance, Moreno-Bote et al. (2007) proposed two different models to explain bi-stable phenomena, with population activity representing perception in both models but with different levels of complexity and spatial scales. The spatial scale of one model was larger (population of neurons vs. spike activity of single neurons), and its complexity level was lower than that of the other (a single equation vs. a set of differential equations); however, both models explained the same psychophysical features. In perspective, the spatial scale and complexity of the models showed a wide range of possibilities, but in almost all models, the representation of percept was still the level of activity of a population of neurons.

**TABLE 1:** Models of visual bi-stable perception.

	Neural model	Model unit	Physiology	Psychophysics
Moreno-Bote et al., 2007 (M1)	Population network and energy minimization	Neural population firing rate	Lateral Inhibition Connections depending on type of neuron and percept	Gamma distribution for dominance durations Levelt II Levelt IV
Moreno-Bote et al., 2007 (M2)	Spiking Neural network, noisy conductance and excitatory-inhibitory connections	Membrane potential	Lateral Inhibition Recurrent excitatory connections Connections depending on type of neuron and percept population	Gamma distribution for dominance durations Levelt II Levelt IV
Wilson, 2003 (M1)	Spike-frequency adaptation produced by slow after-hyperpolarizing potentials	Neuron Firing Rate	Lateral inhibition Adaptation Connections depending on hierarchical model	Swapping binoculary Levelt II
Wilson, 2003 (M2)	Simplified conductance-based model	Membrane Potential	Detailed neural model Adaptation Lateral inhibition Connections depending on hierarchical model	Swapping binoculary Levelt II
Noest et al., 2007	Two pools of neurons, with membrane potential model and an elastic equation	Membrane potential	Adaptation Cross-inhibition (between populations) Gain control by third neuron pool Connections depending on population	Alternation after short interruption (“priming”) Repetition after long interruption (“habituation”)
Wilson et al., 2001	Spike rate network, excitatory-inhibitory populations and wave propagation (local stimulus)	Neuron spike rate	Adaptation Lateral – inhibition (inhibition between adjacent columns) Colinear facilitation Connections depending on: distance, type of neuron and population	Wave propagation
Laing and Chow, 2002	Neural population and Hodgins–Huxley equations	Membrane potential and conductance	Spike frequency adaptation (due to a calcium dependent potassium current) Slow hyperpolarizing current Recurrent excitation Lateral inhibition Excitatory input emulates receptive field (response decay over distance)	Dominance durations Lack of correlations between length of successive events Levelt II Similar stimuli increase mean dominance durations
Freeman, 2005	Hierarchical “box/channel” model of firing rates and post-synaptic potentials	Hierarchical stages of visual pathway	Adaptation Lateral Inhibition Hierarchical connections	Increasing depth of rivalry at higher cortical areas gamma distribution of duration No correlation between dominance durations Differences in eye stimuli implies differences in eye suppression
Gershman et al., 2012	Gibbs Sampling (Markov Chain Monte Carlo)	Abstract representation, which most simplified form can be understood as neural population	Retinotopic map Simplified model, as Wilson (2003)	Gamma distribution Traveling waves Binocular fusion Levelt II
Moreno-Bote et al., 2011	Population network and Energy minimization	Neural population firing rate	Lateral inhibition Connection depending on type of neuron Recurrent excitation	Fraction of dominance follows a Bayesian multiplicative rule
Watanabe et al., 2014	Energy maximization in an energy landscape	Activation of a network of brain regions	Activation of a particular brain area	Mean durations, Frequency of transitions
Cao et al., 2021	Neural population and Ehrenfest process	Proportion of active units	Lateral Inhibition Recurrent excitatory connections Feedback and feedforward connections	Gamma distribution (scaling properties) Levelt I Levelt II Levelt III Levelt IV Positive correlation between successive dominance durations

*References: Laing and Chow, 2002; Wilson, 2003; Freeman, 2005; Moreno-Bote et al., 2007; Noest et al., 2007; Moreno-Bote et al., 2011; Gershman et al., 2012; Cao et al., 2021.*

Despite the variety of models described in the literature, there is still no single model that explains all the psychophysical evidence; furthermore, none of them consider the brain as a whole interactive system. This is partially explained because, so far, models depict specific aspects of multi-stable perception by considering particular features, as they are needed to reproduce the dynamics. Moreover, how the brain represents conscious perception is still debated, so the selection of relevant brain features is somehow arbitrary, as we do not know if they are in fact needed to build perception or if they are just correlated phenomena. In fact, the most basic building blocks for perception are still unknown, as recent evidence stresses the importance of considering a smaller spatial scale such as dendritic organization (Jia et al., 2010) and dendritic action potentials (Moors et al., 2017) in brain computations. These limitations have led to simpler questions, such as the basic brain features needed for multi-stability to occur (for instance, adaptation or noise in Moreno-Bote et al., 2007). Similarly, it is unknown whether perceptual representation is anchored in a local population or in distributed brain areas, which may challenge current models to incorporate distant neural populations and top-down influences, as has been suggested (Hohwy et al., 2008; Braun and Mattia, 2010). This would require the incorporation of a mechanism for the long-range coordination of neurons and populations, as the incorporation of distant areas would require a mechanism for making computations at proper time scales (Varela et al., 2001). More recent studies, have advanced in the aforementioned direction, by predicting all four Levelt laws (Cao et al., 2021) while at the same time incorporating two brain hierarchical levels possibly embodying feedback and feedforward connections (Cao et al., 2016, 2021).

### Network Activity Underlying Multi-Stable Perception

So far, the evidence in humans shows that complex interactions between frontal, parietal, occipital, and temporal areas underlie the dynamics of the duration and changes between the different perceptions (summarized in Box 1; for a review: Brascamp et al., 2018). Despite the large number of areas involved in multi-stable perception, it is not clear whether all areas participate in this dynamic to the same extent or even if their activity is actually related to multi-stability or just to other cognitive processes such as attention, decision making, expectation, motor planning and execution (Frässle et al., 2014). One of the key components of the networks studied so far is the right superior parietal lobule (SPL) (Williams et al., 2003; Carmel et al., 2010; Baker et al., 2015; Megumi et al., 2015), which is an area consistently associated with perceptual transition (Brascamp et al., 2018).

**Box 1. Visual pathways involved in bi-stability.**

Bi-stable perception involves mainly visual areas but also higher areas, enacting top–down modulations. The lateral geniculate nucleus (**LGN**) has yielded contradictory results, with spike activity in non-human primates being unmodulated during rivalry (Lehky and Maunsell, 1996), whereas the fMRI BOLD response correlates with the perceptual state (Haynes et al., 2005; Wunderlich et al., 2005; Schneider, 2009). This contradiction was partially solved when Wilke et al. (2009) showed that slow LFP oscillations but not spike rates at the LGN correlated with the subject’s perception (Wilke et al., 2009). The same study also showed that **pulvinar** spike activity correlated with perception. In **the primary visual cortex**, the V1 spike rate in nonhuman primates correlated with subject perception (Leopold and Logothetis, 1996, 1999; Wilke et al., 2006; Keliris et al., 2010), whereas in humans, the BOLD response (Polonsky et al., 2000; Lee et al., 2005; Zou et al., 2016) and MEG activity (Parkkonen et al., 2008) also covaried with perception. Higher on the visual hierarchy, the **extrastriate visual cortex** showed a clear correlation between functional activity and the dominant percept on fMRI experiments (Lumer et al., 1998; Polonsky et al., 2000) and on intracortical recordings in non-human primates (Logothetis and Schall, 1989; Leopold and Logothetis, 1996; Maier et al., 2008), serving as a good predictor of what will be perceived when the stimulus appears (Tong et al., 1998). Single-unit and LFP recordings in non-human primates also show perceptual modulations at the IT (inferior temporal lobe) (Sheinberg and Logothetis, 1997), MT (medial temporal lobe) (Bradley et al., 1998; Dodd et al., 2001; Grunewald et al., 2002) and STS (superior temporal sulcus) (Logothetis and Schall, 1989).

**Frontal** and **parietal** cortices are also modulated during bi-stability tasks (Sterzer and Rees, 2008), even before the report of the perceptual change (Wang et al., 2013). However, no-report paradigms show reduced frontal activity (Frässle et al., 2014; Brascamp et al., 2015a), whereas subliminal stimulation induces no frontal or parietal modulation (Zou et al., 2016). This suggests that associative areas participate in bi-stability but in an indirect manner. On the other hand, a considerable amount of evidence relates the parietal cortex with perceptual transition, although the extent of its involvement and its precise relation with other areas is still debated (Kanai et al., 2011; Weilnhammer et al., 2013; Baker et al., 2015; Megumi et al., 2015; Roy et al., 2017). Several interpretations have been proposed, including stabilization of the percept (Leopold and Logothetis, 1999; Sterzer and Rees, 2008), feedback error signals in predictive coding paradigms (Hohwy et al., 2008; Brascamp et al., 2018) or the higher level of a network of hierarchical nested attractors (Braun and Mattia, 2010). In any case, it is already clear that an extensive network including structures such as the LGN and pulvinar, V1 and extrastriate cortices all the way up to the frontal and parietal cortices is simultaneously involved in the processing of bi-stable perception.

The role of the SPL in bi-stability was first explained by two distinct functional regions, the anterior SPL (aSPL) and posterior SPL (pSPL) (Carmel et al., 2010; Kanai et al., 2010, 2011); however, recent evidence points out that its role actually comes from different brain networks in which these regions belong (Kanai et al., 2010; Watanabe et al., 2014; Baker et al., 2015; Megumi et al., 2015). For example, Baker et al. (2015) shown that the duration of perception correlate with the activity of three networks including either the aSPL or the pSPL. One of them included the aSPL and the striatum, a second one included the aSPL striatum and the premotor cortex, and a third included the pSPL and the temporal and frontal associative areas. Specifically, using BOLD activity these authors showed that short perceptual stabilities had negative correlations with the functional connectivity between the aSPL and the striatum. Longer perceptual stabilities showed positive correlations with functional connectivity between the aSPL and premotor cortex (on the Necker cube). The functional connectivity of the pSPL and temporal and frontal associative areas showed a positive correlation with perceptual stability (on binocular rivalry).

Other authors using different analytical approaches have also observed that aSPL and pSPL participate in larger networks that correlate with multistable perception (Kanai et al., 2010; Watanabe et al., 2014). Watanabe et al. (2014) found three “basins” or networks operating during bi-stable perception: a visual basin composed of the aSPL, the LOC and V5; an intermediate basin composed only of the FEF; and a frontal basin composed of the pSPL, the FEF and the a/pDLPFC (Watanabe et al., 2014). In this work, the authors related the time that one network stayed dominant with the duration of the percepts. The visual network, which included the aSPL, had a positive correlation with perceptual changes, whereas the frontal network, which included the pSPL, had a negative correlation with the duration of perception (structure from motion; Watanabe et al., 2014). Using a different approach, Megumi et al. (2015) studied temporal correlations among the aPSL, pSPL and V5. This allowed them to determine that the aSPL and pSPL interaction and that the connectivity from the V5 to the pSPL and from the pSPL to the aSPL (but not in the opposite directions) correlated with perceptual durations during bistability. These results highlight that perceptual bi-stability is governed by a long-range bidirectional network integrating sensory and associative areas, including pSPL and aSPL as key structures. The evidence just reviewed came mostly from fMRI studies and, due to its sampling limitations, accounts for very slow temporal oscillations (0.01–4 Hz); nonetheless, there is an ample range of brain processes that also occurs in higher frequency ranges. Increasing evidence suggests that these higher frequency oscillations allow for large-scale coordination in the brain and account for several brain functions, such as attention, perception, and memory (Varela et al., 2001; Buzsáki and Draguhn, 2004; Fries, 2015). In this context, the work from Hipp et al. (2011) shed light on the activity of brain networks at this finer temporal scale (Hipp et al., 2011). They showed, using EEG recordings in human subjects, that two moving bars, perceived as either bouncing or passing each other, correlated with synchronization networks in beta (approximately 15–30 Hz) and gamma bands (above 30 Hz). The beta band network involves the extrastriate visual areas and association areas, specifically areas related to the FEF, PPC (including the IPS and LOC), and the medial extrastriate visual cortex, whereas the gamma networks involve the posterior and medial areas. This suggests that other areas, such as the frontal, parietal and temporal cortices, are involved in bi-stable perception at these higher frequencies (as will be discussed in the next section), and reinforce the idea that bistability may be regulated by network activity.

### Are These Networks Causally Related to Bistability?

Despite the extensive literature relating bi-stability with several brain areas in humans and other primates (Table 2), there are only a few studies directly showing that a particular area is necessary for some bi-stable processes (transitions and stabilities). This evidence comes from TMS studies and through case studies of brain lesions or mental diseases in which a particular brain area or function was altered. TMS evidence has been recently reviewed (Brascamp et al., 2018) and suggests a central role of the SPL (as already discussed in previous paragraphs), which is also the most targeted region in these studies, whereas only a few studies have targeted human middle temporal areas (Brascamp et al., 2010) or frontal areas (de Graaf et al., 2011; Vernet et al., 2015). For instance, Brascamp et al. (2010) applied TMS pulses over the motion sensitive area hMT which resulted in a long-term stabilization of perceptual bias toward the preferred orientation in a structure from motion task, while in absence of TMS pulses, the perceptual bias slowly disappears. Thus, hMT seem to be involved in a long-term buildup of bi-stable perceptual memory. Vernet et al. (2015) showed that a TMS pulse over the IPS decreased percept stability and that this effect was not significant if after this pulse, in a precisely timed manner, a DLPFC pulse was also paired (Vernet et al., 2015). This suggests that frontal areas participate in a larger network controlling the dynamics of bi-stability. Similarly, de Graaf et al. (2011) showed that frontal TMS stimulation impaired the ability to voluntarily control the rate of change in bi-stable perception. Consistent with these results, patients suffering from schizophrenia have also impaired their ability to voluntarily control the rate of change in perception (McBain et al., 2011). This might be caused by the functional dysconnectivity between distant brain areas that characterize this disease (Friston, 1999; Uhlhaas and Singer, 2010). Regarding the few recent studies of brain lesions and bi-stability (Bonneh et al., 2004; Windmann et al., 2006), only one of them addressed the issue of whether the ability to voluntarily increase the rate of change was impaired. Windmann et al. (2006) showed that patients suffering from a lesion on the PFC could not speed up the rate of change compared with controls, although they could sustain the percept as much as controls when they were asked to maintain a particular perception. This evidence is consistent with the involvement of frontal areas in the voluntary control of bi-stable processes. This evidence also supports the idea that long-range network activity is essential for bi-stability. Nevertheless, apart from TMS studies, the amount of research addressing the causal role of different brain areas during bi-stability is scarce.

**TABLE 2:** Brain areas from different organism are modulated by bi-stable stimuli.

Brain area	References	Stimulus	Measures	Subject
V1 and Extrastriate	Wilke et al., 2006	Generalized flash suppression	LFP and MUA	nhp
	Parkkonen et al., 2008	Rubin vase	MEG	Human
	Maier et al., 2008	Generalized flash suppression	fMRI, LFP and SU	nhp
	Leopold and Logothetis, 1996	BR	SU	nhp
	Lee et al., 2005	Traveling waves	fMRI	Human
	Zou et al., 2016	Invisible BR	fMRI	Human
	Keliris et al., 2010	Binocular flash suppression	LFP and SU	nhp
	Gail et al., 2004	BR	LFP & MUA	nhp
	de Jong et al., 2012	Structure from motion	fMRI	Human
	Polonsky et al., 2000	BR	fMRI	Human
	Lumer et al., 1998	BR	fMRI	Human
LGN	Lehky and Maunsell, 1996	BR	SU	nhp
	Wunderlich et al., 2005	BR	fMRI	Human
	Haynes et al., 2005	BR	fMRI	Human
	Schneider, 2009	BR	fMRI	Human
	Wilke et al., 2009	Generalized flash suppression	LFP and SU	nhp
Pulvinar	Wilke et al., 2009	Generalized flash suppression	LFP and SU	nhp
Temporal (IT, MT, SST)	Logothetis and Schall, 1989	BR	SU	nhp
	Bradley et al., 1998	Structure from motion	SU	nhp
	Dodd et al., 2001	Structure from motion	SU	nhp
	Grunewald et al., 2002	Structure from motion	SU	nhp
	Maris and Oostenveld, 2007	Flash suppression	SU	nhp
	Wang et al., 2008	Structure from motion	LFP	nhp
	Sheinberg and Logothetis, 1997	BR and flash suppression	SU	nhp
FFA vs. PPA	Tong et al., 1998	BR (houses vs. faces)	fMRI	Human
Parietal	Kanai et al., 2011	SFM	fMRI + TMS	Human
	Roy et al., 2017	BR	fMRI + EEG	Human
	Williams et al., 2003	Apparent motion	SU	nhp
	Megumi et al., 2015	Structure From Motion	fMRI	Human
Fronto-Parietal	Weilnhammer et al., 2013	Lissajous figure	fMRI	Human
	Lumer et al., 1998	BR	fMRI	Human
Pre-frontal (LPFC)	Sterzer and Rees, 2008	BR	fMRI	Human
	Panagiotaropoulos et al., 2012	Flash suppression	LFP, MUA, and SU	nhp
	Wang et al., 2013	Necker cube, Rubin vase	fMRI	Human
	Frässle et al., 2014	BR without report	fMRI	Human
	Brascamp et al., 2015a	BR	fMRI	Human
FEF	Libedinsky and Livingstone, 2011	Motion induced blindness	SU	nhp
ACC, SMA y PRE-SMA	Gelbard-Sagiv et al., 2018	BR	SU	Human
Network	Hipp et al., 2011	Bounce or pass stimulus	EEG	Human
	Watanabe et al., 2014	Structure from motion	fMRI	Human
	Baker et al., 2015	BR and Necker cube	fMRI	Human
Not visual pathway	Kreiman et al., 2002	Flash Suppression	SU	Human

As reviewed so far, during bi-stability, two essential processes simultaneously occur: on one hand, different and distant brain areas participate in the process, probably embodying a variety of complementary functions; on the other hand, there is a characteristic dynamic of the process, requiring proper coordination between these different functions and their underlying brain processes. Thus, we propose that a neural synchronization mechanism is essential for bi-stability because different functions, subserved by distant regions, such as the parietal, frontal, and occipital areas, must be coordinated in a brief period of time.

## Neural Synchrony in Bi-Stable Perception

### Time-Resolved Approaches Are Necessary for the Study of Bi-Stable Perception

The evidence discussed above supports the hypothesis that the bi-stable process requires neural coordination over distant brain areas (see Box 2). Evidence gathered via fMRI suggests the involvement of different distant brain areas, but fMRI is too slow to reveal the fast temporal dynamics of neural coordination during bi-stable perception. By contrast, single unit activity and LFP data reveal fast and precise synchronization of neural populations during bi-stable perception but only locally inside a very small area surrounding an electrode. Taken together, fMRI and LFP evidence suggests that during bi-stability, local and long-range synchronization occurs within short temporal windows, arguably shorter than the latencies observed at the behavioral level. To try to simultaneously reveal both the brain areas and their coordination during short temporal intervals, the use of the MEEG techniques seem to be the best option, overcoming the low temporal resolution of fMRI and the excessive locality of LFP.

**Box 2. Measures of brain synchrony.**

The study of brain synchrony relies on two complementary mathematical tools: time-based measures and spectral analysis. Time-based measures involve mainly statistical descriptors such as correlations and event-related potentials (ERPs) or event-related fields (ERFs) (Picton et al., 2000; Maris and Oostenveld, 2007). Event-related activity is a highly used method to assess local synchronies at a high temporal resolution because of its advantage when compared with neuroimaging techniques (Nunez and Srinivasan, 2006). In addition, MEG has a high spatial resolution. On the other hand, spectral analysis looks at the data from its frequency content (Kay and Marple, 1981) and incorporates measures of the power and/or phase of the signal at specific frequencies. Power activity is related to local synchronies that are either evoked or induced by stimulus onset (Tallon-Baudry and Bertrand, 1999), whereas phase values are mainly used to assess connectivity between brain areas. Examples of phase measures are the phase-locking value (Lachaux et al., 1999, 2000), coherence, the imaginary part of coherency (Nolte et al., 2004), pairwise phase consistency (Rosenberg et al., 1998), and mean vector length (Fisher, 1995), among others (Bastos and Schoffelen, 2016). Most of the measures mentioned are bivariate methods; they are restricted to studying the relations between the signals from two electrodes and are susceptible to volume conduction confounding factors, especially when applied to EEG data.

MEEG activity has been proposed to reveal both local and long-range transient neural coordination by means of increased oscillations and phase synchronization, respectively (Varela et al., 2001). The following section presents a general definition of synchrony and the main findings regarding neural synchronization and bi-stability.

### Neural Coordination

**FIGURE 2:**
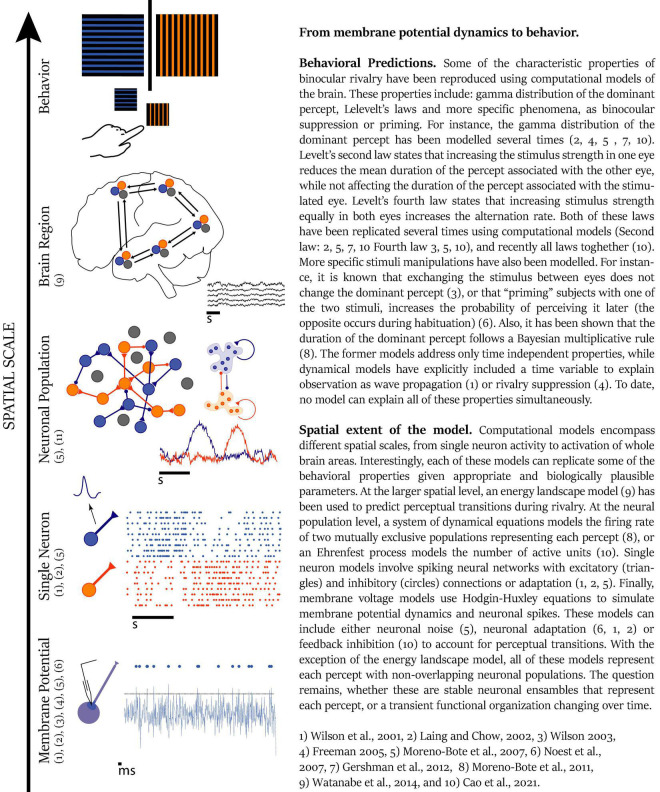
From membrane potential dynamics to behaviour. Reference: 1, Wilson et al. (2001); 2, Laing and Chow (2002); 3, Wilson (2003); 4, Freeman (2005); 5, Moreno-Bote et al. (2007); 6, Noest et al. (2007); 7, Gershman et al. (2012); 8, Moreno-Bote et al. (2011); 9, Watanabe et al. (2014): and 10) Cao et al. (2021).

During the perception of an image, several brain areas engage as part of the same perception process, while the activity of other areas is suppressed or ignored. As discussed in Box 1, the primary visual areas, extrastriate areas, parietal cortex, and frontal cortex should work together in a window of a few tens of milliseconds to generate perception. However, it is not clear how these distant neural populations collaborate in such a brief period of time. One of the mechanisms proposed for brain coordination is time correlation between areas (von der Malsburg, 1994), especially through neural synchrony (Varela, 1995; Singer, 1999; Varela et al., 2001). This theoretical perspective proposes that anatomically distant neuronal populations establish transient connections, forming a closed system or cell assembly for a brief period (Figure 2), and the reverberant activity of this cell assembly has been proposed as the basis for cognitive tasks (Hebb, 1949; Buzsáki and Draguhn, 2004). As an example, during perception, the brain must be able to bind together the relevant features and to segregate the nonrelated features of the whole. When we are faced with a big orange letter T composed of small duplicates of the letter x, we can see the big T, fixate on one small letter x or even just see a pattern of lines. On this examples, different features need to be integrated and segregated in different ways to originate each of these three different perceptions, even though the physical stimulus is the same all the time and the brain areas involved should also be the same, as on feature attention (Ramalingam et al., 2013). To assess unified perception, the areas involved in the construction of the percept should have strong reciprocal connections for a short period of time, thus forming a “cell assembly,” a functional unit of the nervous system (Hebb, 1949).

However, how do cell assemblies achieve their computational tasks? To date, evidence shows that the brain uses two coding dimensions to fulfill its computational demands: space and time (von der Malsburg et al., 2010). The space dimension is used in the representation of incoming sensory information by means of topographically organized (for instance retinotopic or tonotopic) neural activity, thus giving rise to the distributed nature of cortical activity (Quiroga et al., 2005). The time dimension is used as a way to establish brief reciprocal connections between distant populations (von der Malsburg, 1994; Varela, 1995; Varela et al., 2001) in periods as brief as 10 ms, as suggested by behavioral (Goodale et al., 1986) and electrophysiology experiments (Maldonado et al., 2008). These are the two dimensions that the brain uses to bind and segregate features and to move from the current to the next brain state.

Neural synchrony has been proposed as a general mechanism to bind features in a unified percept (Uhlhaas et al., 2009). Neural synchrony has been observed in visual tasks, including Mooney faces (Rodriguez et al., 1999), word recognition (Melloni et al., 2007), and oddball tasks (Brázdil et al., 2013), as well as in somatosensory (Palva et al., 2005), auditory (Dykstra et al., 2011), and multisensory modalities (Senkowski et al., 2008). In a case of visual perception, Mooney faces presented for brief periods elicited a transient increase in long range (Rodriguez et al., 1999) and local gamma-band synchrony (Grützner et al., 2010). These experiments showed that synchronization occurred when the different elements of the stimuli were integrated in a unified percept. To investigate whether synchronization was related to the moment of perception of the stimulus and not only to the binding process, Melloni et al. (2007) used a detection task. Subjects viewed a masked visual stimulus (a word) that could be perceived only in a fraction (close to 50%) of the trials. They found an increase in long-range gamma-band synchrony for perceived words compared with unperceived words. Palva et al. (2005) showed that in an equivalent somatosensory task, there was also more synchrony when subjects were able to detect a tactile stimulus, in this case in the alpha band. These examples suggest that the increase in neural synchrony correlates with the emerging perception resulting from the binding process and not with the processing of stimuli parts or subthreshold stimuli parts.

### Event-Related Potential and Bi-Stable Perception

Among MEEG analyses, the most commonly used method to study brain-evoked responses in cognitive sciences is event-related potential (ERP) analysis. ERPs provide is a continuous and temporally precise measure of brain processes with low spatial resolution (Luck, 2005). Many of the experiments involving ERP on bi-stable perception use discontinuous presentation methods (Kornmeier and Bach, 2004, 2005, 2006, 2009, 2012; Pitts et al., 2008) to compare evoked responses either between perceptual transitions and stabilities or between endogenous and exogenous transitions. The first approach provides insight into the brain mechanism under perceptual changes, whereas the second approach provides insight into the self-generated processes that lead to spontaneous changes in perception. Regarding comparisons between perceptual transition and stability, the most consistent ERP difference found in several publications is reversal negativity (RN) (Kornmeier and Bach, 2004, 2005, 2006, 2009, 2012; Pitts et al., 2008; Britz et al., 2009; Intaite et al., 2010). A negative ERP deflection peaking at approximately 300 ms after stimulus presentation occurs in both exogenously and endogenously triggered changes in perception (40 ms later for endogenously triggered changes than for exogenously triggered changes). ERPs are related to the changes in perception as its amplitude changes between stability and transitions. Pitts et al. (2008) also found that ERP amplitude increases when subjects try to reverse perception voluntarily. To further study reversal negativity, Intaite et al. (2010) adjusted the intensity of the perceptual experience using two arrays of cubes, one on each hemifield of the screen, to manipulate the subject’s attention and awareness levels. In this discontinuous presentation task, they found that the RN is specifically correlated with changes in perception (Intaite et al., 2010) and not with changes in attention or in the level of awareness. Recently Joos et al. (2020) showed that ERP observed after perceptual transitions resemble the effect after Gestalt construction in non-bi-stable stimuli. This result indicates that part of the effects observed so far reflect the process of perceptual disambiguation and may not be exclusive of bi-stable phenomena. Future experiments should address (1) whether reversal negativity is observable when viewing other bi-stable stimuli because Kornmeier and Bach (2014) did not observe RN activity when using Boring’s old/young woman stimulus and (2) whether the RN is still present under continuous paradigms (Kornmeier and Bach, 2014). The results presented indicate that the RN is present on endogenously and exogenously induced transitions, reflecting changes in perception during the bi-stability process, and that it can be modulated endogenously (Pitts et al., 2008). Importantly, this ERP already occurs 250 ms after stimulus onset, supporting the need for time-resolved methods such as MEEG.

As has been described in fMRI experiments, endogenous vs. exogenous comparisons have already revealed brain areas that are particularly activated when changes in perception occur spontaneously, without any change in the stimulus. For instance, de Jong et al. (2012) have shown that external sensory repetition (exogenous) attenuates BOLD activity on visual areas, while perceptual repetition (endogenous) enhances BOLD activity on early visual areas, ventral visual stream (V4 and LO) and parietal areas, suggesting a network activation for perception as compared to mere sensation processing (de Jong et al., 2012). The ERP literature has also addressed the differences between endogenous versus exogenous transitions to reveal different temporal dynamics between self-produced or externally triggered changes in perception. Kornmeier and Bach (2009) analyzed ERPs to endogenous and exogenous transitions using two different bi-stable images, the Necker cube and Boring’s old/lady woman. They found ERP differences of approximately 400 ms between these two conditions; the exogenous condition had a higher amplitude and duration than the endogenously triggered ERP. Specifically, on endogenously evoked transitions, the peaks of the ERPs occur 40–70 ms later than in exogenously triggered transitions (Kornmeier and Bach, 2006). These results show that the dynamics of the ERPs between the two conditions (exogenous and endogenous changes) are different, with endogenous transitions requiring more time to develop.

As evident in fMRI scans, EEG source localization signals the role of the right parietal cortex on endogenous transitions. Ongoing activity has been proposed to reflect endogenous brain processes (Raichle, 2010). Britz et al. (2009) studied the role of ongoing activity in endogenous perceptual changes using a discontinuous presentation of Necker cubes. They found that increased activity in the right parietal cortex 50 ms before stimulus presentation correlated with perceptual reversal. Only a few experiments have also used TMS stimulation to further assess the causal role of previous brain activity on multi-stable perception (Vernet et al., 2015). These authors showed that TMS stimulation over the intraparietal sulcus before stimuli presentation decreased the stability of the percept and modulated the ERP over the right parietal cortex. Both results confirm a parietal role during bi-stability on EEG recordings, as shown for fMRI scans. This finding also supports a role of the frontal α-band in this phenomenon. Together, these results suggest different and possibly opposing roles for each brain region, but more evidence is needed to precisely understand the effect of stimulation on this brain process.

### Slow Oscillations (δ and α Activity)

As suggested in the previous sections, changes in perception relate to previous brain activity, with slow oscillation modulation appearing up to 1000 ms before subject reports. In a line-moving paradigm, in which the subject perceived either four independent lines or a diamond, Flevaris et al. (2013) found a decrease in α-band activity before the button press. Specifically, for both percepts the decrement in α-band power start 1000 ms before a button press at occipital sites. It was especially low when switching from perceiving lines to perceiving a diamond (Flevaris et al., 2013). In an EEG-fMRI experiment of a continuous presentation of the Necker cube, Ozaki et al. (2012) showed a modulation of δ-band power spreading from frontal to parietal areas before the subject’s report of a perceptual change. The spectral power at 3–4 Hz increased in the left frontal and right centroparietal electrodes. This activity sequentially peaked at 750, 600, and 350 ms before the subject’s report along the dorsal attentional network. From these two observations, the authors suggested that the slow oscillation initiated traveling activity along the dorsal path (Ozaki et al., 2012). For another moving stimulus, Haendel and Jensen showed that α-band lateralization preceded the onset of illusory perception (Händel and Jensen, 2014). They used a variation of the MIB task in which they superimposed a moving grating at each hemifield. Subjects reported when gratings spontaneously disappeared and appeared. They found via MEG that a spontaneous grid appearance correlated with early α activity, in good agreement with EEG studies previously reviewed (Ozaki et al., 2012; Flevaris et al., 2013).

Together, this evidence suggests that (1) several areas peaked in a traveling pattern from frontal to occipital regions and (2) these different brain processes developed on a short time scale (<1 s).

### Beta Band Activity

Increments in β-band activity correlate with spontaneous changes in perception and are usually interpreted as a top–down modulation of brain activity (Kloosterman et al., 2015). In a continuous perceptual task of apparent motion, in which the subject’s perception changes from real movement to the apparent direction of movement, VanRullen et al. (2006) showed that the main difference in EEG recordings was at the β-band, approximately 13 Hz. The modulation in β-band power during changes in perception had a right centroparietal distribution and its dynamics were related to the reported perception. It decreased 1.5 s before transitions from real to illusory movement and increases in transitions from illusory to real movement (VanRullen et al., 2006). In accordance with these results, Kloosterman et al. (2015) found a decrease in β-band activity associated with the illusory disappearance of a target stimulus during a motion-induced blindness task and an increase in the same band during its reappearance. The authors showed that the amplitude of β-band suppression predicted the duration of the associated perceptual illusion, which, along with several controls, led them to suggest that the activity during disappearance was a top–down modulation. The main difference between the two studies was the location of the β activity; Kloosterman et al. (2015) found a clearly occipital topography, whereas VanRullen et al. (2006) found centroparietal activity. This could be explained by the difference in the acquisition system, considering that the former used MEG whereas the latter used EEG, and the differences in paradigms between the two studies.

β-band activity seems to play several roles in top–down processes. Zaretskaya and Bartels (2015) found that bi-stable stimuli with global and local gestalts elicited β-band reductions before the onset of the global percept. Global states were associated with a lower β-band power in comparison with the local states (Zaretskaya and Bartels, 2015). This suggests a possible role of the β-band in global binding. In addition, beta band activity is modulated during changes in perception. In an intermittent-presentation task with EEG recordings, Yokota et al. (2014) found that spontaneous perceptual reversals correlated with increases in β-band activity (16–36 Hz) in the right occipital regions compared with the activity in response to stability. This activity had an early component (100–150 ms), probably related to the disambiguation process, and a late component (350–450 ms), which has been interpreted as the correlate of the conscious processing of perception. Both experiments are consistent with a top–down function associated with the β-band but also show that this band may reflect distinct functions depending on the task.

### Gamma Band Activity

Gamma band activity has shown a more diverse relation with bi-stable perception than other frequency bands. On one hand, gamma band activity has been observed preceding changes in perception of frontal areas, as previously discussed. On the other hand, it has also been interpreted as reflecting inhibitory processes on occipital cortices.

It has been reported that an increase in the γ-band at frontal electrodes precedes changes in the perception of rivalry (Doesburg et al., 2009; Händel and Jensen, 2014) in ambiguous perception tasks (Ehm et al., 2011). This activity occurs before reversals (Ehm et al., 2011) and is associated with voluntary manipulation by the subject of the perception duration (Mathes et al., 2006). In the former case (Ehm et al., 2011), the authors suggested that the induced γ-band activity might reflect a state of maximal instability of the brain that would lead to the consequent reversal of perception. This result agrees with the hypothesis that a frontoparietal network controls transition during bi-stability. In the latter case, frontal γ-band activity is enhanced when subjects voluntarily maintain perception, suggesting a role of the γ-band in top–down modulation (Mathes et al., 2006), consistent with frontal lesions that disrupt voluntary control of the duration of perception (Windmann et al., 2006).

### Role of Synchrony on Different Frequency Bands During Bi-Stability

**FIGURE 3:**
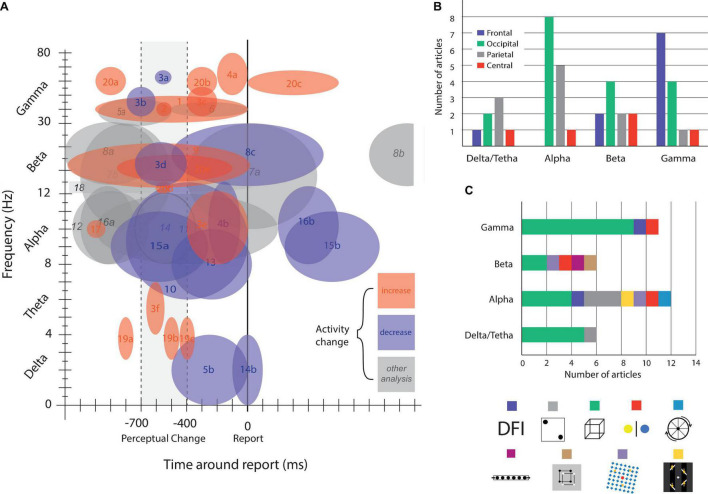
Brain activity modulation by time, frequency, and area. **(A)** Brain oscillatory activity modulation related to bi-stable perception. Numbers indicate references and letters brain areas or other commentaries. **(B)** Articles published on bi-stable perception grouped by oscillatory frequency band and colored by brain area. **(C)** Number of published articles grouped by frequency band, colored by bi-stable stimulus. References: (1) Basar-Eroglu et al. (1996); (2) Doesburg et al. (2009); (3) Ehm et al. (2011), [a] frontal, [b] occipital, [c] central, [d] parietal, frontal, central, [e] occipital, parietal, [f] occipital; (4) Lange et al. (2013) [a] gamma power correlates with subjective perception, [b] alpha power inversely correlate with subjective perception; (5) Mathes et al. (2006), [a] enhanced for hold condition, [b] a decrease in delta wave around this time window is observed; (6) Strüber and Herrmann (2002); (7) Kloosterman et al. (2015), [a] decrease for illusory disappearance and increase for reappearance, [b] decrease before reappearance; (8) Piantoni et al. (2010), [a] higher for veridical percept, [b] decrease for BR and Moving dots illusion around report; (9) Zaretskaya and Bartels (2015), beta decreases more for local percept. (10) Basar-Eroglu et al. (2016), alpha power was even more decreased in patients with schizophrenia. (11) Flevaris et al. (2013), there were more decrease for object percept compared with fragment percept; (12) Händel and Jensen (2014), there is a significant alpha lateralization preceding the estimated illusory disappearance of the stimuli, the level of lateralization predicts the duration of the following illusion; (13) Isoglu-Alkaç and Strüber (2006), alpha band activity was lower in the interval between 500 and 1000 ms before report than 0–500 ms before; (14) Mathes et al. (2010), [a] alpha power is higher on parietal and occipital electrodes for standard report, compared with delayed one, [b] a decrease in delta wave is observed around perceptual change (in standard and delayed conditions); (15) Piantoni et al. (2017), [a] alpha power start decreasing 900 ms before report, reaching it minimum at 250 ms, [b] after 250 ms alpha power start increasing until 850 ms after report (reaching starting levels). (16) Piantoni et al. (2010), [a] veridical percept show higher alpha activity, [b] after report of both veridical and illusory percept alpha power decreases; (17) Strüber and Herrmann (2002), the decrease in activity was not observed for exogenous induced changes; (18) VanRullen et al. (2006); (19) Ozaki et al. (2012), [a] frontocentral, [b] parietal, [c] central parietal; (20) Devia et al. (2020), [a] frontal, parietal, occipital, [b] parietal-occipital, [c] parietal-occipital, [d] frontal, [e] parietal-occipital, [f] occipital; (21) Yokota et al. (2014).

As shown in Figure 3A, different frequency bands coexist during bi-stable processes. The coexistence and interaction of different frequency bands is a well-known phenomenon occurring in the brain (Varela, 1995; Varela et al., 2001; VanRullen and Koch, 2003; Jensen and Colgin, 2007). Varela in his 1995 work proposed that if at a given time 2 or more neural assemblies were competing to be established as a unified cognitive experience, these neural assemblies should be expressed as having different spatiotemporal patterns; therefore, the dynamics of neural synchrony could be reflected in different frequencies (Varela, 1995; Varela et al., 2001; VanRullen and Koch, 2003; Pockett et al., 2009; Schroeder and Lakatos, 2009; Uhlhaas et al., 2009).

Many hypotheses have been formulated for the role of synchrony in different frequency bands. One such hypothesis is the frequency based on topography (Varela, 1995; von der Malsburg et al., 2010). This idea proposes that synchrony between distant neuronal populations, or within a large population, is mediated by slow bands (θ and β), whereas local oscillations involve fast frequency bands (mainly γ). Another hypothesis, proposed by VanRullen and Koch (2003), is that frequency bands act as a mechanism of multiplexed representations in visual perception. It proposes that during explicit visual perception, cortical oscillations at two different frequency bands, one slow and the other fast, constitute the neuronal ‘context’ and ‘content’, respectively (for example: α and γ bands in the visual system). This interaction between different frequencies could be the basis for a process of discrete perception, consistent with psychophysics results (VanRullen, 2016). In a similar way, Uhlhaas et al. (2009) expressed the need to study the coexistence of oscillations in different bands, their interactions, their temporal organization and coordination between them, since they could encode nested relations in frequency. Hypothetically, these nested relations serve not only the representation of objects but also compound movements (Uhlhaas et al., 2009). Schroeder and Lakatos (2009) presented other hypotheses regarding the role of oscillations in different frequency bands. They proposed a hierarchical organization of the oscillations, which would control the baseline excitability and thus the response associated with a stimulus (Lakatos et al., 2005; Schroeder and Lakatos, 2009). These ideas are not mutually exclusive and are probably complementary given that complex cognitive processes involve interactions spanning through multiple bands.

There are only a few papers directly addressing the role of multiple bands on bi-stability. It has been reported that the power in the δ-band increases whereas the α-band decreases during Necker cube reversal (Isoglu-Alkaç et al., 2000; Isoglu-Alkaç and Strüber, 2006). Additionally, on binocular rivalry, there is an increment in fronto-occipital γ synchronization associated with the subject’s report that is phase-coupled to theta rhythm (Doesburg et al., 2009). Nakatani and van Leeuwen found a cooccurrence of γ- and α-band activity during Necker cube perception. Specifically, there are brief periods of γ synchrony between parietal and frontal areas. These events start 800 to 600 ms before the report of perceptual change with a simultaneous modulation in the α-band at occipital electrodes (Nakatani and van Leeuwen, 2006). Additionally, in a double flash illusion task, γ-power is enhanced and α-band power decreases before reporting perception, and in both cases, the magnitude of power modulation correlates with subject perception (Lange et al., 2013). Together, these results could be interpreted as (1) that the simultaneous modulation of the δ, α, and γ bands reflect the interaction between different frequencies reflecting one of the mechanisms previously mentioned or (2) that the modulation of specific frequencies co-occurred during the task, reflecting different local brain processes. Regardless of the interpretation, the evidence reviewed so far supports that multiple oscillations coexist during bi-stability (Figure 3A) in different brain regions (Figure 3B), with a compendium of frequency band modulations suggesting the coordination of multiple brain areas following a specific temporal and spatial pattern of activation (Figure 3A) in preferred frequency bands (Figure 3B). It also shows that despite being a single phenomenon, bi-stability can entail specific brain oscillations depending on the experimental design (Figure 3C). Some frequency dynamics are almost exclusively found in Necker cube experiments (gamma and delta/theta bands), whereas others (especially the alpha band) seem to be more ubiquitously present under different experimental conditions. Finally, the evidence reveals a complex process that develops in no more than 1 s, reinforcing the need to study multiple brain regions at the proper spatial and time scales. Further research should shed light on whether the interactions between these oscillations are an exclusive component of bi-stability dynamics or a general mechanism to coordinate distant and local brain regions in a short period of time.

## Discussion: Why Integrating Modeling, fMRI and MEG Evidence Is Difficult and Why Such Integration Is Necessary

**FIGURE 4:**
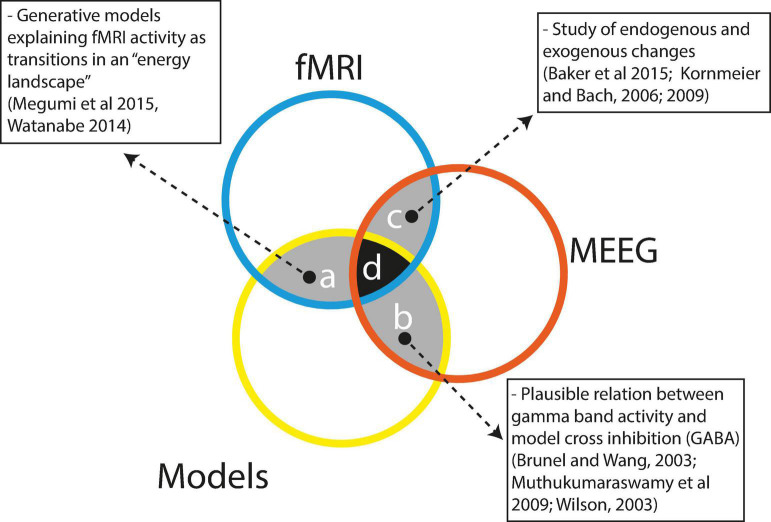
Scarce integration between the techniques currently used to study bi-stable perception. References: Megumi et al. (2015); Watanabe et al. (2014); Baker et al. (2015); Kornmeier and Bach (2006, 2009); Brunel and Wang (2003); Muthukumaraswamy et al. (2009); Wilson (2003).

Multi-stable perception is a widely studied area with a large body of evidence arising from modeling, fMRI and MEEG studies; nevertheless, as seen in Figure 4, integrated research is still very scarce. Here, we will argue why such evidence is difficult to integrate and why it is essential to try to integrate it.

Findings from modeling studies are difficult to integrate with findings from fMRI/MEEG studies because these approaches represent opposite perspectives on how to uncover the neural bases of multi-stable perception. fMRI/MEEG studies involve a bottom-up strategy, as experiments are performed to try to determine which brain areas and processes are active during multi-stable perception, trying to collect the pieces of the puzzle without an *a priori* idea on how such pieces should fit together into the mechanism of multi-stable perception. By contrast, modeling studies display a top–down research strategy, starting with a clear idea of what the mechanism giving rise to multi-stable perception should be. The modeler proceeds to choose which parameters are critical to her or his model and to fine tune the values of the parameters to obtain a behavior similar to the modelized phenomenon. This is done by concentrating on a few biologically plausible parameters while disregarding all the remaining biological processes pertaining to multi-stable perception.

Thus, modeling studies disregard most of the complexity of biological phenomena, whereas experimental studies fail to propose a clear mechanism by which neural activity produces bi-stable perception. Because of these opposing perspectives, both research programs so far have engaged in insufficient crosstalk and synergetic interactions.

On the other hand, given that both fMRI and MEEG approaches share a common experimental perspective, it would be reasonable to expect that data produced by fMRI/MEEG studies would be easier to integrate. Data comparison, however, has proven to be challenging (Ritter et al., 2009) because both techniques are sensitive to different brain structures and physiological processes. Concerning brain regions, fMRI is capable of detecting activities all over the brain, including deep subcortical structures, whereas MEEG is mostly sensitive to superficial cortical brain activity. From a physiological perspective, both techniques also differ, with MEEG recording fast electromagnetic activity, mostly dendritic postsynaptic potentials of pyramidal cells, whereas fMRI records a slow increase in oxygenated blood supply to the active brain areas. As a result of these differences, experiments comparing MEEG and fMRI data must integrate restrictions imposed by each technique (Britz and Pitts, 2011); even so, when comparing results, we should be aware that the sources of the described brain activity may not intersect.

Do we need to try to integrate such information? The necessity for integrating research arises from the complexity of multi-stable perception. Experimental research has shown the involvement of a multiplicity of different brain areas activated in specific temporal patterns involving several frequency bands. These brain regions, patterns and frequencies are modulated depending on the specific stimulus and the task involved, suggesting not a single but rather a set of related neural mechanisms. This complexity should be embraced by studies aspiring to realistically represent the process of multi-stable perception.

Some efforts have been made to integrate the different approaches. As depicted in Figure 4, region a, modeling studies have integrated information from fMRI research as in the model by Watanabe et al. (2014), in which the authors integrated fMRI information to show that a model with three attraction basins representing visual area states, frontal area states and intermediate area states appropriately could describe the dynamics of perceptual stability and change during structure from motion bi-stable perception. The authors nicely show that, as predicted by the model, subjects with greater frontal activity display faster changes than subjects with activity predominantly in visual areas. In a similar way, Megumi et al. (2015) used fMRI information and dynamic causal modeling to construct a model with three interacting areas: r-V5, r-pSPL, and r-aSPL; this model correctly described the dynamics of residence and change during a structure from a motion perceptual paradigm. In this study, the authors were able to show that the strength of bottom-up connections (r-V5 ->r-pSPL - > r-aSPL) predicted the stability of perception.

Models integrating MEEG information are almost completely lacking with only indirect evidence (Figure 4, region b), suggesting a link between gamma activity, GABA levels, lateral inhibition and reversion rates in bi-stable perception. Evidence has related peak gamma activity with occipital GABA levels that would be responsible for the observed bi-stable switch ratios (Brunel and Wang, 2003; Muthukumaraswamy et al., 2009). This interpretation is consistent with computational models of adaptation and horizontal inhibition (Wilson, 2003; Tong et al., 2006; Kang and Blake, 2010). In a classical binocular rivalry task, Fries (2015) showed that the peak amplitude of evoked γ-band activity in V1 observed in a detection task was inversely correlated with the perceptual switch ratio in a binocular task (using the same stimuli). Previous evidence correlated the peak frequency of induced γ-band activity with resting levels of GABA (Muthukumaraswamy et al., 2009) and GABA levels with perceptual switch ratios (van Loon et al., 2013). This evidence led authors to interpret the peak frequency as an index of neuronal population inhibition. Consistently, in the case of perceptual rivalry, perceptual alterations are explained by inhibitory connections in the visual cortex, the activity of which would be reflected by GABA levels.

Finally, as depicted in Figure 4, region c, under specific conditions, some MEEG and fMRI studies have yielded converging results. Given the low time resolution of fMRI, only relatively slow processes can be used to compare fMRI and MEEG. Particularly the comparison between endogenously versus exogenously driven bi-stable perception has been suitable. Both fMRI (Baker et al., 2015) and MEEG (Kornmeier and Bach, 2009) studies have pointed to the involvement of right parietal and frontal cortices, with frontal and parietal cortices performing antagonistic functions. Additionally, EEG (Kornmeier and Bach, 2006) and iEEG (de Jong et al., 2020) data have shown smaller and delayed potentials in endogenously driven bi-stable perception, suggesting that the brain takes longer to ‘make up its mind’ when self-organizing than when processing external stimulation. Also, de Jong et al. (2020) have shown using iEEG that external stimulation is processed with neural activity starting at V1 and then proceeding to the higher ventral stream cortices. By contrast, endogenous self-organized perception starts in higher ventral cortices and then proceeds backward to primary visual cortices. Additionally, delta and alpha MEEG oscillations appear to be slow enough to be compared with fMRI data. Ozaki et al. (2012) showed a traveling delta wave involving the dorsal attentional network by successive activations of left frontal, right parietal and centroparietal electrodes. However, as the preceding studies involved different tasks and stimulations, caution is advised in interpreting the results.

As seen in Figure 4 (regions a, b, and c) pairwise interactions between fMRI, MEEG and models do exist; however, the challenge of integrating the three of them has not been undertaken so far. This is hardly surprising given the difficulties that must be surpassed to successfully complete research involving simultaneous MEEG recordings, fMRI scans and modeling of multi-stable perception. We believe that a research program particularly relevant for the understanding of multi-stable perception would involve modeling studies including multi-level brain interactions mediated by multi-frequency oscillatory dynamics. However, is the gain worth the effort? We believe so because multi-stable perception is a particular instantiation of more general functions of human cognition. Self-organization, self-determination and self-control are all crucial characteristics of mind/brain autonomy because studying their properties may provide us with hints and intuition as to whether a materially determined system can achieve freedom.

## Open Questions (Future Experiments)

**OQ: Where does the ‘perception’ of bi-stable perception take place?** Evidence so far shows that bi-stable perception is represented along several stages of the visual pathways (Box 1); nevertheless, we still do not know which, if any, of these areas are sufficient for bi-stable perception.

**OQ: When does the ‘perception’ of bi-stable perception take place?** MEEG studies show that bi-stable perception develops by a series of parallel activations involving different frequency bands in different brain regions. However, it is still unknown when perception emerges. Perhaps complementary measures, such as pupil dynamics, heart rate variability, or eye movements, are needed to solve this issue.

**OQ: How can we progress from correlational to causal evidence on bi-stable perception?** To date, the computational models that reproduce the dynamics of bi-stable perception assume the alternation of two mutually exclusive neural populations. However, current evidence is mostly correlational, so further experiments are needed to learn whether such activity is sufficient for perception.

**OQ: How does long-range neural synchronization participate in bi-stable perception models?** Given the spatially distributed activity shown on fMRI scans and the fast dynamics evidenced by MEEG, long-range synchronization is a plausible mechanism to fulfill both requirements. However, we still do not know how to incorporate this property into current models.

**OQ: How does voluntary perceptual change occur?** The current explanation for endogenous changes in perception is either noise (introduced by perceptual processing or due to background brain activity) or neural adaptation; nevertheless, this does not explain voluntary changes in perception.

## Author Contributions

CD and MC-M review the literature. CD, MC-M, and ER discussed the ideas and wrote the manuscript. All authors contributed to the article and approved the submitted version.

## Conflict of Interest

The authors declare that the research was conducted in the absence of any commercial or financial relationships that could be construed as a potential conflict of interest.

## Publisher’s Note

All claims expressed in this article are solely those of the authors and do not necessarily represent those of their affiliated organizations, or those of the publisher, the editors and the reviewers. Any product that may be evaluated in this article, or claim that may be made by its manufacturer, is not guaranteed or endorsed by the publisher.

## Glossary

**Multi-stable perception:** (or Perceptual Rivalry) Perception in which two or more interpretations of the same stimulus alternate involuntarily. When only two perceptions are possible, the term bi-stable perception is preferred. Two exemplary experimental paradigms are binocular rivalry and bi-stable stimuli.

**Latent Variable:** The parameters or physiological features that are supposed to participate in the process that leads to perception and alternations but that are not strictly necessary for them.

**Complexity Level:** The number and type of interactions, in time and space, between the different features that modulate and constrain the variable representing perception; these values are typically resumed in a set of differential equations.

**Spatial Scale of a model:** Based on Varela et al.’s (2001) definition, this dimension refers to the spatial scale that the model is built on, including sub-neuron features such as dendrites, ionic conductance or population activity.

## References

[B1] Andrews T. J. Schluppeck D. Homfray D. Matthews P. Blakemore C. (2002). Activity in the fusiform gyrus predicts conscious perception of Rubin’s vase-face illusion. *NeuroImage* 17 890–901. 10.1006/nimg.2002.1243 12377163

[B2] Baker D. H. Karapanagiotidis T. Coggan D. D. Wailes-Newson K. Smallwood J. (2015). Brain networks underlying bistable perception. *Neuroimage* 119 229–234. 10.1016/j.neuroimage.2015.06.053 26123379

[B3] Basar-Eroglu C. Mathes B. Khalaidovski K. Brand A. Schmiedt-Fehr C. (2016). Altered alpha brain oscillations during multistable perception in schizophrenia. *Int. J. Psychophysiol.* 103 118–128. 10.1016/j.ijpsycho.2015.02.002 25746892

[B4] Basar-Eroglu C. Strüber D. Kruse P. Başar E. Stadler M. (1996). Frontal gamma-band enhancement during multistable visual perception. *Int. J. Psychophysiol.* 24 113–125. 10.1016/s0167-8760(96)00055-4 8978438

[B5] Bastos A. M. Schoffelen J.-M. (2016). A tutorial review of functional connectivity analysis methods and their interpretational pitfalls. *Front. Syst. Neurosci.* 9:175. 10.3389/fnsys.2015.00175 PMC4705224 26778976

[B6] Blake R. Logothetis N. K. (2002). Visual competition. *Nat. Rev. Neurosci.* 3 13–21. 11823801 10.1038/nrn701

[B7] Bonneh Y. S. Pavlovskaya M. Ring H. Soroker N. (2004). Abnormal binocular rivalry in unilateral neglect: evidence for a non-spatial mechanism of extinction. *Neuroreport* 15 473–477. 10.1097/00001756-200403010-00018 15094506

[B8] Bradley D. C. Chang G. C. Andersen R. A. (1998). Encoding of three-dimensional structure-from-motion by primate area MT neurons. *Nature* 392 714–717. 10.1038/33688 9565031

[B9] Brascamp J. W. Kanai R. Walsh V. van Ee R. (2010). Human middle temporal cortex, perceptual bias, and perceptual memory for ambiguous three-dimensional motion. *J. Neurosci.* 30 760–766. 10.1523/JNEUROSCI.4171-09.2010 20071541 PMC6633007

[B10] Brascamp J. W. Klink P. C. Levelt W. J. M. (2015b). The ‘laws’ of binocular rivalry: 50 years of Levelt’s propositions. *Vis. Res.* 109 20–37. 10.1016/j.visres.2015.02.019 25749677

[B11] Brascamp J. W. Blake R. Knapen T. (2015a). Negligible fronto-parietal BOLD activity accompanying unreportable switches in bistable perception. *Nat. Neurosci.* 18 1672–1678. 10.1038/nn.4130 26436901 PMC4603386

[B12] Brascamp J. W. Sterzer P. Blake R. Knapen T. (2018). Multistable perception and the role of the frontoparietal cortex in perceptual inference. *Annu. Rev. Psychol.* 69 77–103. 28854000 10.1146/annurev-psych-010417-085944

[B13] Braun J. Mattia M. (2010). Attractors and noise: twin drivers of decisions and multistability. *Neuroimage* 52 740–751. 10.1016/j.neuroimage.2009.12.126 20083212

[B14] Brázdil M. Janecek J. T. Klimes P. Marecek R. Roman R. Jurak P. (2013). On the time course of synchronization patterns of neuronal discharges in the human brain during cognitive tasks. *PLoS One* 8:e63293. 10.1371/journal.pone.0063293 PMC3655978 23696809

[B15] Britz J. Pitts M. A. (2011). Perceptual reversals during binocular rivalry: ERP components and their concomitant source differences. *Psychophysiology* 48 1490–1499. 10.1111/j.1469-8986.2011.01222.x 21668451

[B16] Britz J. Landis T. Michel C. M. (2009). Right parietal brain activity precedes perceptual alternation of bistable stimuli. *Cereb. Cortex* 19 55–65. 10.1093/cercor/bhn056 18424780

[B17] Brunel N. Wang X.-J. (2003). What determines the frequency of fast network oscillations with irregular neural discharges? I. Synaptic dynamics and excitation-inhibition balance. *J. Neurophysiol.* 90 415–430. 10.1152/jn.01095.2002 12611969

[B18] Buzsáki G. Draguhn A. (2004). Neuronal oscillations in cortical networks. *Science* 304 1926–1929. 15218136 10.1126/science.1099745

[B19] Cao R. Pastukhov A. Aleshin S. Mattia M. Braun J. (2021). Binocular rivalry reveals an out-of-equilibrium neural dynamics suited for decision-making. *Elife* 10:e61581. 10.7554/eLife.61581 34369875 PMC8352598

[B20] Cao R. Pastukhov A. Mattia M. Braun J. (2016). Collective activity of many bistable assemblies reproduces characteristic dynamics of multistable perception. *J. Neurosci.* 36 6957–6972. 10.1523/JNEUROSCI.4626-15.2016 27358454 PMC6604901

[B21] Carmel D. Walsh V. Lavie N. Rees G. (2010). Right parietal TMS shortens dominance durations in binocular rivalry. *Curr. Biol.* 20 R799–R800. 10.1016/j.cub.2010.07.036 20869603

[B22] Crick F. Koch C. (1990). Some reflections on visual awareness. *Cold Spring Harb. Symp. Quant. Biol.* 55 953–962. 10.1101/sqb.1990.055.01.089 2132872

[B23] de Graaf T. A. de Jong M. C. Goebel R. van Ee R. Sack A. T. (2011). On the functional relevance of frontal cortex for passive and voluntarily controlled bistable vision. *Cereb. Cortex* 21 2322–2331. 10.1093/cercor/bhr015 21385836

[B24] de Jong M. C. Kourtzi Z. Ee R. (2012). Perceptual experience modulates cortical circuits involved in visual awareness. *Eur. J. Neurosci.* 36 3718–3731. 10.1111/ejn.12005 23031201 PMC7611163

[B25] de Jong M. C. Vansteensel M. J. van Ee R. Leijten F. S. S. Ramsey N. F. Dijkerman H. C. (2020). Intracranial recordings reveal unique shape and timing of responses in human visual cortex during illusory visual events. *Curr. Biol.* 30 3089–3100.e4. 10.1016/j.cub.2020.05.082 32619489

[B26] Devia C. Rodriguez E. Maldonado P. E. (2020). *Brief Periods Of Gamma Band Enhancement Correlate With Sustain Perception.* Manuscript in preparation

[B27] Dodd J. V. Krug K. Cumming B. G. Parker A. J. (2001). Perceptually bistable three-dimensional figures evoke high choice probabilities in cortical area MT. *J. Neurosci.* 21 4809–4821. 10.1523/JNEUROSCI.21-13-04809.2001 11425908 PMC6762355

[B28] Doesburg S. M. Green J. J. McDonald J. J. Ward L. M. (2009). Rhythms of consciousness: binocular rivalry reveals large-scale oscillatory network dynamics mediating visual perception. *PLoS One* 4:e6142. 10.1371/journal.pone.0006142 PMC2702101 19582165

[B29] Dykstra A. R. Halgren E. Thesen T. Carlson C. E. Doyle W. Madsen J. R. (2011). Widespread brain areas engaged during a classical auditory streaming task revealed by intracranial EEG. *Front. Hum. Neurosci.* 5:74. 10.3389/fnhum.2011.00074 PMC3154443 21886615

[B30] Ehm W. Bach M. Kornmeier J. (2011). Ambiguous figures and binding: EEG frequency modulations during multistable perception. *Psychophysiology* 48 547–558. 10.1111/j.1469-8986.2010.01087.x 20796247

[B31] Einhäuser W. Martin K. A. C. König P. (2004). Are switches in perception of the Necker cube related to eye position? *Eur. J. Neurosci.* 20 2811–2818. 10.1111/j.1460-9568.2004.03722.x 15548224

[B32] Einhäuser W. Stout J. Koch C. Carter O. (2008). Pupil dilation reflects perceptual selection and predicts subsequent stability in perceptual rivalry. *Proc. Natl. Acad. Sci. U.S.A.* 105 1704–1709. 10.1073/pnas.0707727105 18250340 PMC2234208

[B33] Engel A. K. Singer W. (2001). Temporal binding and the neural correlates of sensory awareness. *Trends Cogn. Sci.* 5 16–25. 10.1016/s1364-6613(00)01568-0 11164732

[B34] Fisher N. I. (1995). *Statistical Analysis Of Circular Data.* Cambridge: Cambridge University Press.

[B35] Flevaris A. V. Martínez A. Hillyard S. A. (2013). Neural substrates of perceptual integration during bistable object perception. *J. Vis.* 13:17. 10.1167/13.13.17 24246467 PMC3833463

[B36] Frässle S. Sommer J. Jansen A. Naber M. Einhäuser W. (2014). Binocular rivalry: frontal activity relates to introspection and action but not to perception. *J. Neurosci.* 34 1738–1747. 10.1523/JNEUROSCI.4403-13.2014 24478356 PMC6827584

[B37] Freeman A. W. (2005). Multistage model for binocular rivalry. *J. Neurophysiol.* 94 4412–4420. 10.1152/jn.00557.2005 16148271

[B38] Fries P. (2015). Rhythms for cognition: communication through coherence. *Neuron* 88 220–235. 10.1016/j.neuron.2015.09.034 26447583 PMC4605134

[B39] Friston K. J. (1999). Schizophrenia and the disconnection hypothesis. *Arch. Gen. Psychiatry* 99 68–79. 10.1111/j.1600-0447.1999.tb05985.x 10225335

[B40] Gail A. Brinksmeyer H. J. Eckhorn R. (2004). Perception-related modulations of local field potential power and coherence in primary visual cortex of awake monkey during binocular rivalry. *Cereb. Cortex* 14 300–313. 14754869 10.1093/cercor/bhg129

[B41] Gelbard-Sagiv H. Mudrik L. Hill M. R. Koch C. Fried I. (2018). Human single neuron activity precedes emergence of conscious perception. *Nat. Commun.* 9:2057. 10.1038/s41467-018-03749-0 PMC5970215 29802308

[B42] Gershman S. J. Vul E. Tenenbaum J. B. (2012). Multistability and perceptual inference. *Neural Comput.* 24 1–24. 10.1162/NECO_a_00226 22023198

[B43] Goodale M. A. Pelisson D. Prablanc C. (1986). Large adjustments in visually guided reaching do not depend on vision of the hand or perception of target displacement. *Nature* 320 748–750. 10.1038/320748a0 3703000

[B44] Grunewald A. Bradley D. C. Andersen R. A. (2002). Neural correlates of structure-from-motion perception in macaque V1 and MT. *J. Neurosci.* 22 6195–6207. 10.1523/JNEUROSCI.22-14-06195.2002 12122078 PMC6757912

[B45] Grützner C. Uhlhaas P. J. Genc E. Kohler A. Singer W. Wibral M. (2010). Neuroelectromagnetic correlates of perceptual closure processes. *J. Neurosci.* 30 8342–8352. 10.1523/JNEUROSCI.5434-09.2010 20554885 PMC6634569

[B46] Händel B. F. Jensen O. (2014). Spontaneous local alpha oscillations predict motion-induced blindness. *Eur. J. Neurosci.* 40 3371–3379. 10.1111/ejn.12701 25174681

[B47] Hasson U. Hendler T. Bashat D. B. Malach R. (2001). Vase or face? A neural correlate of shape-selective grouping processes in the human brain. *J. Cogn. Neurosci.* 13 744–753. 10.1162/08989290152541412 11564319

[B48] Haynes J.-D. Deichmann R. Rees G. (2005). Eye-specific effects of binocular rivalry in the human lateral geniculate nucleus. *Nature* 438 496–499. 10.1038/nature04169 16244649 PMC1351280

[B49] Hebb D. O. (1949). *The Organization Of Behavior: A Neuropsychological Theory.* Hoboken, NY: Wiley.

[B50] Hipp J. F. Engel A. K. Siegel M. (2011). Oscillatory synchronization in large-scale cortical networks predicts perception. *Neuron* 69 387–396. 10.1016/j.neuron.2010.12.027 21262474

[B51] Hohwy J. Roepstorff A. Friston K. (2008). Predictive coding explains binocular rivalry: an epistemological review. *Cognition* 108 687–701. 10.1016/j.cognition.2008.05.010 18649876

[B52] Intaite M. Koivisto M. Ruksenas O. Revonsuo A. (2010). Reversal negativity and bistable stimuli: attention, awareness, or something else? *Brain Cogn.* 74 24–34. 10.1016/j.bandc.2010.06.002 20598419

[B53] Isoglu-Alkaç Ü Basar-Eroglu C. Ademoglu A. Demiralp T. Miener M. Stadler M. (2000). Alpha activity decreases during the perception of Necker cube reversals: an application of wavelet transform. *Biol. Cybern.* 82 313–320. 10.1007/s004220050585 10804063

[B54] Isoglu-Alkaç U. Strüber D. (2006). Necker cube reversals during long-term EEG recordings: sub-bands of alpha activity. *Int. J. Psychophysiol.* 59 179–189. 10.1016/j.ijpsycho.2005.05.002 16023748

[B55] Jensen O. Colgin L. L. (2007). Cross-frequency coupling between neuronal oscillations. *Trends Cogn. Sci.* 11 267–269. 10.1016/j.tics.2007.05.003 17548233

[B56] Jia H. Rochefort N. L. Chen X. Konnerth A. (2010). Dendritic organization of sensory input to cortical neurons in vivo. *Nature* 464 1307–1312. 10.1038/nature08947 20428163

[B57] Joos E. Giersch A. Hecker L. Schipp J. Heinrich S. P. Tebartz van Elst L. (2020). Large EEG amplitude effects are highly similar across Necker cube, smiley, and abstract stimuli. *PLoS One* 15:e0232928. 10.1371/journal.pone.0232928 PMC7239493 32433672

[B58] Kanai R. Bahrami B. Rees G. (2010). Human parietal cortex structure predicts individual differences in perceptual rivalry. *Curr. Biol.* 20 1626–1630. 10.1016/j.cub.2010.07.027 20727757 PMC2949566

[B59] Kanai R. Carmel D. Bahrami B. Rees G. (2011). Structural and functional fractionation of right superior parietal cortex in bistable perception. *Curr. Biol.* 21 R106–R107. 10.1016/j.cub.2010.12.009 21300270 PMC3084447

[B60] Kang M.-S. Blake R. (2010). What causes alternations in dominance during binocular rivalry? *Atten. Percept. Psychophys.* 72 179–186. 10.3758/APP.72.1.179 20045887 PMC2811269

[B61] Kay S. M. Marple S. L. (1981). Spectrum analysis—A modern perspective. *Proc. IEEE* 69 1380–1419.

[B62] Keliris G. A. Logothetis N. K. Tolias A. S. (2010). The role of the primary visual cortex in perceptual suppression of salient visual stimuli. *J. Neurosci.* 30 12353–12365. 10.1523/JNEUROSCI.0677-10.2010 20844131 PMC2962415

[B63] Klink P. C. van Ee R. Nijs M. M. Brouwer G. J. Noest A. J. van Wezel R. J. (2008). Early interactions between neuronal adaptation and voluntary control determine perceptual choices in bistable vision. *J. Vis.* 8 16.1–18. 10.1167/8.5.16 18842087

[B64] Kloosterman N. A. Meindertsma T. Hillebrand A. van Dijk B. W. Lamme V. A. Donner T. H. (2015). Top-down modulation in human visual cortex predicts the stability of a perceptual illusion. *J. Neurophysiol.* 113 1063–1076. 10.1152/jn.00338.2014 25411458 PMC4329440

[B65] Kogo N. Kern F. B. Nowotny T. van Ee R. van Wezel R. Aihara T. (2021). Dynamics of a mutual inhibition circuit between pyramidal neurons compared to human perceptual competition. *J. Neurosci.* 41:1251. 10.1523/JNEUROSCI.2503-20.2020 33443089 PMC7888225

[B66] Kornmeier J. Bach M. (2004). Early neural activity in Necker-cube reversal: evidence for low-level processing of a gestalt phenomenon. *Psychophysiology* 41 1–8. 10.1046/j.1469-8986.2003.00126.x 14692995

[B67] Kornmeier J. Bach M. (2005). The Necker cube—an ambiguous figure disambiguated in early visual processing. *Vis. Res.* 45 955–960. 10.1016/j.visres.2004.10.006 15695180

[B68] Kornmeier J. Bach M. (2006). Bistable perception – along the processing chain from ambiguous visual input to a stable percept. *Int. J. Psychophysiol.* 62 345–349. 10.1016/j.ijpsycho.2006.04.007 16808985

[B69] Kornmeier J. Bach M. (2009). Object perception: when our brain is impressed but we do not notice it. *J. Vis.* 9 7.1–10. 10.1167/9.1.7 19271877

[B70] Kornmeier J. Bach M. (2012). Ambiguous Figures – what happens in the brain when perception changes but not the stimulus. *Front. Hum. Neurosci.* 6:51. 10.3389/fnhum.2012.00051 22461773 PMC3309967

[B71] Kornmeier J. Bach M. (2014). EEG correlates of perceptual reversals in Boring’s ambiguous old/young woman stimulus. *Perception* 43 950–962. 10.1068/p7741 25420334

[B72] Kreiman G. Fried I. Koch C. (2002). Single-neuron correlates of subjective vision in the human medial temporal lobe. *Proc. Natl. Acad. Sci. U.S.A.* 99 8378–8383. 12034865 10.1073/pnas.072194099 PMC123075

[B73] Lachaux J. P. Rodriguez E. Le Van Quyen M. Lutz A. Martinerie J. Varela F. J. (2000). Studying single-trials of phase synchronous activity in the brain. *Int. J. Bifurcation Chaos (IJBC)* 10 2429–2439. 10.1142/s0218127400001560

[B74] Lachaux J. P. Rodriguez E. Martinerie J. Varela F. J. (1999). Measuring phase synchrony in brain signals. *Hum. Brain Mapp.* 8 194–208. 10.1002/(sici)1097-0193(1999)8:4<194::aid-hbm4>3.0.co;2-c 10619414 PMC6873296

[B75] Laing C. R. Chow C. C. (2002). A spiking neuron model for binocular rivalry. *J. Comput. Neurosci.* 12 39–53. 10.1023/a:1014942129705 11932559

[B76] Lakatos P. Shah A. S. Knuth K. H. Ulbert I. Karmos G. Schroeder C. E. (2005). An oscillatory hierarchy controlling neuronal excitability and stimulus processing in the auditory cortex. *J. Neurophysiol.* 94 1904–1911. 10.1152/jn.00263.2005 15901760

[B77] Lange J. Oostenveld R. Fries P. (2013). Reduced occipital alpha power indexes enhanced excitability rather than improved visual perception. *J. Neurosci.* 33 3212–3220. 10.1523/JNEUROSCI.3755-12.2013 23407974 PMC6619207

[B78] Lee S.-H. Blake R. Heeger D. J. (2005). Traveling waves of activity in primary visual cortex during binocular rivalry. *Nat. Neurosci.* 8 22–23. 10.1038/nn1365 15580269 PMC1215464

[B79] Lehky S. R. Maunsell J. H. R. (1996). No binocular rivalry in the LGN of alert macaque monkeys. *Vis. Res.* 36 1225–1234. 10.1016/0042-6989(95)00232-4 8711902

[B80] Leopold D. A. Logothetis N. K. (1996). Activity changes in early visual cortex reflect monkeys’ percepts during binocular rivalry. *Nature* 379 549–553. 10.1038/379549a0 8596635

[B81] Leopold D. A. Logothetis N. K. (1999). Multistable phenomena: changing views in perception. *Trends Cogn. Sci.* 3 254–264. 10.1016/s1364-6613(99)01332-7 10377540

[B82] Libedinsky C. Livingstone M. (2011). Role of prefrontal cortex in conscious visual perception. *J. Neurosci.* 31 64–69. 21209190 10.1523/JNEUROSCI.3620-10.2011 PMC3079255

[B83] Logothetis N. Schall J. (1989). Neuronal correlates of subjective visual perception. *Science* 245 761–763. 10.1126/science.2772635 2772635

[B84] Luck S. J. (2005). *An Introduction To The Event-Related Potential Technique (Cognitive Neuroscience).* Cambridge, MA: MIT Press.

[B85] Lumer E. D. Friston K. J. Rees G. (1998). Neural correlates of perceptual rivalry in the human brain. *Science* 280 1930–1934. 10.1126/science.280.5371.1930 9632390

[B86] Maier A. Wilke M. Aura C. Zhu C. Ye F. Q. Leopold D. A. (2008). Divergence of fMRI and neural signals in V1 during perceptual suppression in the awake monkey. *Nat. Neurosci.* 11 1193–1200. 10.1038/nn.2173 18711393 PMC2754054

[B87] Maldonado P. Babul C. Singer W. Rodriguez E. Berger D. Grün S. (2008). Synchronization of neuronal responses in primary visual cortex of monkeys viewing natural images. *J. Neurophysiol.* 100 1523–1532. 10.1152/jn.00076.2008 18562559

[B88] Maris E. Oostenveld R. (2007). Nonparametric statistical testing of EEG- and MEG-data. *J. Neurosci. Meth.* 164 177–190. 10.1016/j.jneumeth.2007.03.024 17517438

[B89] Mathes B. Pomper U. Walla P. Basar-Eroglu C. (2010). Dissociation of reversal- and motor-related delta- and alpha-band responses during visual multistable perception. *Neurosci. Lett.* 478 14–18. 10.1016/j.neulet.2010.04.057 20435088

[B90] Mathes B. Strüber D. Stadler M. A. Basar-Eroglu C. (2006). Voluntary control of Necker cube reversals modulates the EEG delta- and gamma-band response. *Neurosci. Lett.* 402 145–149. 10.1016/j.neulet.2006.03.063 16630691

[B91] McBain R. Norton D. J. Kim J. Chen Y. (2011). Reduced cognitive control of a visually bistable image in schizophrenia. *J. Int. Neuropsychol. Soc.* 17 551–556. 10.1017/S1355617711000245 21385517

[B92] Megumi F. Bahrami B. Kanai R. Rees G. (2015). Brain activity dynamics in human parietal regions during spontaneous switches in bistable perception. *Neuroimage* 107 190–197. 10.1016/j.neuroimage.2014.12.018 25512040 PMC4306523

[B93] Melloni L. Molina C. Pena M. Torres D. Singer W. Rodriguez E. (2007). Synchronization of neural activity across cortical areas correlates with conscious perception. *J. Neurosci.* 27 2858–2865. 10.1523/JNEUROSCI.4623-06.2007 17360907 PMC6672558

[B94] Moors P. Hesselmann G. Wagemans J. van Ee R. (2017). Continuous flash suppression: stimulus fractionation rather than integration. *Trends Cogn. Sci.* 21 719–721. 10.1016/j.tics.2017.06.005 28690078

[B95] Moreno-Bote R. Knill D. C. Pouget A. (2011). Bayesian sampling in visual perception. *Proc. Natl. Acad. Sci. U.S.A.* 108 12491–12496. 10.1073/pnas.1101430108 21742982 PMC3145684

[B96] Moreno-Bote R. Rinzel J. Rubin N. (2007). Noise-induced alternations in an attractor network model of perceptual bistability. *J. Neurophysiol.* 98 1125–1139. 10.1152/jn.00116.2007 17615138 PMC2702529

[B97] Muthukumaraswamy S. D. Edden R. A. E. Jones D. K. Swettenham J. B. Singh K. D. (2009). Resting GABA concentration predicts peak gamma frequency and fMRI amplitude in response to visual stimulation in humans. *Proc. Natl. Acad. Sci. U.S.A.* 106 8356–8361. 10.1073/pnas.0900728106 19416820 PMC2688873

[B98] Nakatani H. van Leeuwen C. (2006). Transient synchrony of distant brain areas and perceptual switching in ambiguous figures. *Biol. Cybern.* 94 445–457. 10.1007/s00422-006-0057-9 16532332

[B99] Noest A. J. van Ee R. Nijs M. M. van Wezel R. J. A. (2007). Percept-choice sequences driven by interrupted ambiguous stimuli: a low-level neural model. *J. Vis.* 7:10. 10.1167/7.8.10 17685817

[B100] Nolte G. Bai O. Wheaton L. Mari Z. Vorbach S. Hallett M. (2004). Identifying true brain interaction from EEG data using the imaginary part of coherency. *Clin. Neurophysiol.* 115 2292–2307. 10.1016/j.clinph.2004.04.029 15351371

[B101] Nunez P. L. Srinivasan R. (2006). *Electric Fields Of The Brain: The Neurophysics of EEG.* Oxford: Oxford University Press.

[B102] Orbach J. Zucker E. (1965). Reversibility of the necker cube. VI. Effects of interpolating a non-reversing cube. *Percept. Motor Skill* 20 470–472. 10.2466/pms.1965.20.2.470 14279324

[B103] Orbach J. Ehrlich D. Heath H. A. (1963). Reversibility of the Necker cube. I. An examination of the concept of “satiation of orientation”. *Percept. Motor Skill* 17 439–458. 10.2466/pms.1963.17.2.439 14065532

[B104] Ozaki T. J. Sato N. Kitajo K. Someya Y. Anami K. Mizuhara H. (2012). Traveling EEG slow oscillation along the dorsal attention network initiates spontaneous perceptual switching. *Cogn. Neurodyn.* 6 185–198. 10.1007/s11571-012-9196-y 22511914 PMC3311835

[B105] Palva S. Linkenkaer-Hansen K. Näätänen R. Palva J. M. (2005). Early neural correlates of conscious somatosensory perception. *J. Neurosci.* 25 5248–5258. 10.1523/JNEUROSCI.0141-05.2005 15917465 PMC6724814

[B106] Panagiotaropoulos T. I. Deco G. Kapoor V. Logothetis N. K. (2012). Neuronal discharges and gamma oscillations explicitly reflect visual consciousness in the lateral prefrontal cortex. *Neuron* 74 924–935. 22681695 10.1016/j.neuron.2012.04.013

[B107] Parkkonen L. Andersson J. Hämäläinen M. Hari R. (2008). Early visual brain areas reflect the percept of an ambiguous scene. *Proc. Nat. Acad. Sci. U.S.A.* 105:20500. 10.1073/pnas.0810966105 19074267 PMC2602606

[B108] Piantoni G. Kline K. A. Eagleman D. M. (2010). Beta oscillations correlate with the probability of perceiving rivalrous visual stimuli. *J. Vis.* 10 18–18. 10.1167/10.13.18 21149311

[B109] Piantoni G. Romeijn N. Gomez-Herrero G. Werf Y. D. V. D. Someren E. J. W. V. (2017). Alpha power predicts persistence of bistable perception. *Sci. Rep.* 7:5208. 10.1038/s41598-017-05610-8 28701732 PMC5507912

[B110] Picton T. W. Bentin S. Berg P. Donchin E. Hillyard S. A. Johnson R. Jr. (2000). Guidelines for using human event-related potentials to study cognition: recording standards and publication criteria. *Psychophysiology* 37 127–152. 10.1111/1469-8986.3720127 10731765

[B111] Pitts M. A. Gavin W. J. Nerger J. L. (2008). Early top-down influences on bistable perception revealed by event-related potentials. *Brain Cogn.* 67 11–24. 10.1016/j.bandc.2007.10.004 18155339

[B112] Pockett S. Bold G. E. J. Freeman W. J. (2009). EEG synchrony during a perceptual-cognitive task: widespread phase synchrony at all frequencies. *Clin. Neurophysiol.* 120 695–708. 10.1016/j.clinph.2008.12.044 19250863

[B113] Polonsky A. Blake R. Braun J. Heeger D. J. (2000). Neuronal activity in human primary visual cortex correlates with perception during binocular rivalry. *Nat Neurosci* 3 1153–1159. 10.1038/80676 11036274

[B114] Quiroga R. Q. Reddy L. Kreiman G. Koch C. Fried I. (2005). Invariant visual representation by single neurons in the human brain. *Nature* 435 1102–1107. 10.1038/nature03687 15973409

[B115] Raichle M. E. (2010). Two views of brain function. *Trends Cogn. Sci.* 14 180–190. 10.1016/j.tics.2010.01.008 20206576

[B116] Ramalingam N. McManus J. N. J. Li W. Gilbert C. D. (2013). Top-down modulation of lateral interactions in visual cortex. *J. Neurosci.* 33 1773–1789. 10.1523/JNEUROSCI.3825-12.2013 23365217 PMC3711382

[B117] Ritter P. Moosmann M. Villringer A. (2009). Rolandic alpha and beta EEG rhythms’ strengths are inversely related to fMRI-BOLD signal in primary somatosensory and motor cortex. *Hum. Brain Mapp.* 30 1168–1187. 10.1002/hbm.20585 18465747 PMC6870597

[B118] Rodriguez E. George N. Lachaux J. P. Martinerie J. Renault B. Varela F. J. (1999). Perception’s shadow: long-distance synchronization of human brain activity. *Nature* 397 430–433. 10.1038/17120 9989408

[B119] Rosenberg J. R. Halliday D. M. Breeze P. Conway B. A. (1998). Identification of patterns of neuronal connectivity—partial spectra, partial coherence, and neuronal interactions. *J. Neurosci. Meth.* 83 57–72. 10.1016/s0165-0270(98)00061-2 9765051

[B120] Roy A. V. Jamison K. W. He S. Engel S. A. He B. (2017). Deactivation in the posterior mid-cingulate cortex reflects perceptual transitions during binocular rivalry: Evidence from simultaneous EEG-fMRI. *Neuroimage* 152 1–11. 10.1016/j.neuroimage.2017.02.041 28219776 PMC5531216

[B121] Schneider K. A. (2009). High-resolution imaging of the human thalamus and superior colliculus during binocular rivalry. *J. Vis.* 9 271–271.

[B122] Schroeder C. E. Lakatos P. (2009). Low-frequency neuronal oscillations as instruments of sensory selection. *Trends Neurosci.* 32 9–18. 10.1016/j.tins.2008.09.012 19012975 PMC2990947

[B123] Senkowski D. Schneider T. R. Foxe J. J. Engel A. K. (2008). Crossmodal binding through neural coherence: implications for multisensory processing. *Trends Neurosci.* 31 401–409. 10.1016/j.tins.2008.05.002 18602171

[B124] Sheinberg D. L. Logothetis N. K. (1997). The role of temporal cortical areas in perceptual organization. *Proc. Natl. Acad. Sci. U.S.A.* 94 3408–3413. 10.1073/pnas.94.7.3408 9096407 PMC20383

[B125] Singer W. (1999). Neuronal synchrony: a versatile code for the definition of relations? *Neuron* 24 49–65, 111–125. 10677026 10.1016/s0896-6273(00)80821-1

[B126] Sterzer P. Rees G. (2008). A neural basis for percept stabilization in binocular rivalry. *J. Cogn. Neurosci.* 20 389–399. 10.1162/jocn.2008.20039 18004954

[B127] Sterzer P. Kleinschmidt A. Rees G. (2009). The neural bases of multistable perception. *Trends Cogn. Sci.* 13 310–318. 10.1016/j.tics.2009.04.006 19540794

[B128] Strüber D. Herrmann C. S. (2002). MEG alpha activity decrease reflects destabilization of multistable percepts. *Cogn. Brain Res.* 14 370–382. 10.1016/s0926-6410(02)00139-8 12421660

[B129] Tallon-Baudry C. Bertrand O. (1999). Oscillatory gamma activity in humans and its role in object representation. *Trends Cogn. Sci.* 3 151–162. 10322469 10.1016/s1364-6613(99)01299-1

[B130] Tong F. Meng M. Blake R. (2006). Neural bases of binocular rivalry. *Trends Cogn. Sci.* 10 502–511. 10.1016/j.tics.2006.09.003 16997612

[B131] Tong F. Nakayama K. Vaughan J. T. Kanwisher N. (1998). Binocular rivalry and visual awareness in human extrastriate cortex. *Neuron* 21 753–759. 10.1016/s0896-6273(00)80592-9 9808462

[B132] Tsuchiya N. Wilke M. Frässle S. Lamme V. A. F. (2015). No-report paradigms: extracting the true neural correlates of consciousness. *Trends Cogn. Sci.* 19 757–770. 10.1016/j.tics.2015.10.002 26585549

[B133] Uhlhaas P. J. Singer W. (2010). Abnormal neural oscillations and synchrony in schizophrenia. *Nat. Rev. Neurosci.* 11 100–113. 10.1038/nrn2774 20087360

[B134] Uhlhaas P. J. Pipa G. Lima B. Melloni L. Neuenschwander S. Nikolić D. (2009). Neural synchrony in cortical networks: history, concept and current status. *Front. Integr. Neurosci.* 3:17. 10.3389/neuro.07.017.2009 PMC2723047 19668703

[B135] Van Ee R. van Dam L. C. J. Brouwer G. J. (2005). Voluntary control and the dynamics of perceptual bi-stability. *Vis. Res.* 45 41–55. 10.1016/j.visres.2004.07.030 15571737

[B136] van Loon A. M. Knapen T. Scholte H. S. St John-Saaltink E. Donner T. H. Lamme V. A. (2013). GABA shapes the dynamics of bistable perception. *Curr. Biol.* 23 823–827. 10.1016/j.cub.2013.03.067 23602476

[B137] VanRullen R. (2016). Perceptual cycles. *Trends Cogn. Sci.* 20 723–735. 27567317 10.1016/j.tics.2016.07.006

[B138] VanRullen R. Koch C. (2003). Is perception discrete or continuous? *Trends Cogn. Sci.* 7 207–213. 10.1016/s1364-6613(03)00095-0 12757822

[B139] VanRullen R. Reddy L. Koch C. (2006). The continuous wagon wheel illusion is associated with changes in electroencephalogram power at approximately 13 Hz. *J. Neurosci.* 26 502–507. 10.1523/JNEUROSCI.4654-05.2006 16407547 PMC6674399

[B140] Varela F. J. (1995). Resonant cell assemblies: a new approach to cognitive functions and neuronal synchrony. *Biol. Res.* 28 81–95. 8728823

[B141] Varela F. Lachaux J. P. Rodriguez E. Martinerie J. (2001). The brainweb: phase synchronization and large-scale integration. *Nat. Rev. Neurosci.* 2 229–239. 10.1038/35067550 11283746

[B142] Vernet M. Brem A.-K. Farzan F. Pascual-Leone A. (2015). Synchronous and opposite roles of the parietal and prefrontal cortices in bistable perception: a double-coil TMS-EEG study. *Cortex* 64 78–88. 10.1016/j.cortex.2014.09.021 25461709 PMC4346464

[B143] von der Malsburg C. (1994). “The correlation theory of brain function,” in *Models of Neural Networks. Physics of Neural Networks*, eds Domany E. van Hemmen J. L. Schulten K. (New York, NY: Springer), 95–119. 10.1007/978-1-4612-4320-5_2

[B144] von der Malsburg C. (1999). The what and why of binding: the modeler’s perspective. *Neuron* 24 49–65, 111–125. 10.1016/s0896-6273(00)80825-9 10677030

[B145] von der Malsburg C. Phillips W. A. Singer W. (2010). *Dynamic Coordination In The Brain.* Cambridge, MA: MIT Press.

[B146] Wang M. Arteaga D. He B. J. (2013). Brain mechanisms for simple perception and bistable perception. *Proc. Natl. Acad. Sci. U.S.A.* 110 E3350–E3359. 10.1073/pnas.1221945110 23942129 PMC3761598

[B147] Wang Z. Logothetis N. K. Liang H. (2008). Decoding a bistable percept with integrated time-frequency representation of single-trial local field potential. *J. Neural Eng.* 5 433–442. 10.1088/1741-2560/5/4/008 18971518

[B148] Watanabe T. Masuda N. Megumi F. Kanai R. Rees G. (2014). Energy landscape and dynamics of brain activity during human bistable perception. *Nat. Commun.* 5:4765. 10.1038/ncomms5765 25163855 PMC4174295

[B149] Weilnhammer V. A. Ludwig K. Hesselmann G. Sterzer P. (2013). Frontoparietal cortex mediates perceptual transitions in bistable perception. *J. Neurosci.* 33 16009–16015. 10.1523/JNEUROSCI.1418-13.2013 24089505 PMC6618467

[B150] Wilke M. Logothetis N. K. Leopold D. A. (2006). Local field potential reflects perceptual suppression in monkey visual cortex. *Proc. Natl. Acad. Sci. U.S.A.* 103 17507–17512. 10.1073/pnas.0604673103 17088545 PMC1859959

[B151] Wilke M. Mueller K.-M. Leopold D. A. (2009). Neural activity in the visual thalamus reflects perceptual suppression. *Proc. Natl. Acad. Sci. U.S.A.* 106 9465–9470. 10.1073/pnas.0900714106 19458249 PMC2684842

[B152] Williams Z. M. Elfar J. C. Eskandar E. N. Toth L. J. Assad J. A. (2003). Parietal activity and the perceived direction of ambiguous apparent motion. *Nat. Neurosci.* 6 616–623. 10.1038/nn1055 12730699

[B153] Wilson H. R. (2003). Computational evidence for a rivalry hierarchy in vision. *Proc. Natl. Acad. Sci. U.S.A.* 100 14499–14503. 10.1073/pnas.2333622100 14612564 PMC283620

[B154] Wilson H. R. Blake R. Lee S. H. (2001). Dynamics of travelling waves in visual perception. *Nature* 412 907–910. 10.1038/35091066 11528478

[B155] Windmann S. Wehrmann M. Calabrese P. Güntürkün O. (2006). Role of the prefrontal cortex in attentional control over bistable vision. *J. Cogn. Neurosci.* 18 456–471. 10.1162/089892906775990570 16513009

[B156] Wunderlich K. Schneider K. A. Kastner S. (2005). Neural correlates of binocular rivalry in the human lateral geniculate nucleus. *Nat. Neurosci.* 8 1595–1602. 10.1038/nn1554 16234812 PMC1470662

[B157] Yokota Y. Minami T. Naruse Y. Nakauchi S. (2014). Neural processes in pseudo perceptual rivalry: an ERP and time-frequency approach. *Neuroscience* 271 35–44. 10.1016/j.neuroscience.2014.04.015 24759770

[B158] Zaretskaya N. Bartels A. (2015). Gestalt perception is associated with reduced parietal beta oscillations. *Neuroimage* 112 61–69. 10.1016/j.neuroimage.2015.02.049 25731988

[B159] Zhou Y. H. Gao J. B. White K. D. Merk I. Yao K. (2004). Perceptual dominance time distributions in multistable visual perception. *Biol. Cybern.* 90 256–263. 10.1007/s00422-004-0472-8 15085344

[B160] Zou J. He S. Zhang P. (2016). Binocular rivalry from invisible patterns. *Proc. Natl. Acad. Sci. U.S.A.* 113 8408–8413. 27354535 10.1073/pnas.1604816113 PMC4968763

